# Perioperative immune dynamics during cardiopulmonary bypass and association with major adverse postoperative events

**DOI:** 10.3389/fcvm.2026.1811178

**Published:** 2026-05-26

**Authors:** Zhiyuan Cheng, Xinyi Liao, Juan Wu, Ping Yang, Qinjuan Wu, Zongcheng Tang, Yishun Wang, Wentong Meng, Lei Du, Jing Lin

**Affiliations:** 1 Department of Anesthesiology, West China Hospital, Sichuan University, Chengdu, Sichuan, China; 2 Department of Anesthesiology, Sichuan Cancer Hospital, Chengdu, Sichuan, China; 3 Department of Anesthesiology, Chongqing University Three Gorges Hospital, Wanzhou, Chongqing, China; 4 Department of Anesthesiology, Chengdu Second People's Hospital, Chengdu, Sichuan, China; 5 Laboratory of Stem Cell Biology, State Key Laboratory of Biotherapy, West China Hospital, Sichuan University, Chengdu, Sichuan, China

**Keywords:** cardiopulmonary bypass, flow cytometry, immune dysregulation, organ dysfunction, perioperative inflammation

## Abstract

**Purpose:**

To clarify how cardiopulmonary bypass (CPB) affects perioperative immune cell populations and how these changes are associated with postoperative organ dysfunction.

**Methods:**

We prospectively recruited 60 consecutive patients who underwent CPB as part of elective valvular surgery and/or coronary artery bypass grafting at our center. Peripheral blood samples were collected before surgery, during rewarming, at the end of CPB, and 24 h after surgery. The populations of neutrophils, monocytes, natural killer (NK) cells, T cells, and B cells were analyzed using flow cytometry, and changes were compared between patients with and without major adverse postoperative events (MAEs) within 30 days after surgery. MAEs included acute kidney injury, neurological dysfunction, liver injury, cardiovascular complications, and respiratory dysfunction.

**Results:**

Of the 60 patients analyzed, 10 (16.7%) developed MAEs. CPB was associated with marked perioperative immune changes, with leukocyte and neutrophil counts peaking at 24 h after surgery, whereas lymphocyte counts declined and reached their nadir at the same time point. During rewarming, patients with MAEs had higher proportions of CD56dimCD314⁺ NK cells (96.74 ± 2.41 vs. 88.41 ± 11.75%, *P* = 0.035) and CD284⁺ non-classical monocytes (71.36 ± 17.95 vs. 49.13 ± 22.42%, *P* = 0.006), but lower proportions of CD163⁺ classical monocytes (69.46 ± 27.59 vs. 85.04 ± 15.53%, *P* = 0.035) and CD45RO⁺ T cells (35.62 ± 6.93 vs. 43.22 ± 11.66%, *P* = 0.023). At the end of CPB, patients with MAEs had higher proportions of CD4⁺CD38⁺ T cells (59.16 ± 15.16 vs. 41.78 ± 18.60%, *P* = 0.004) and CD274⁺ unswitched memory B cells (42.69 ± 15.29 vs. 23.80 ± 15.04%, *P* < 0.001). Exploratory receiver operating characteristic curves (ROC) analyses provided descriptive estimates of within-cohort discrimination for several immune subpopulations. In risk-adjusted Firth penalized logistic regression, higher CD284⁺ non-classical monocytes during rewarming [adjusted odds ratio (OR) = 3.118, 95% confidence interval (CI): 1.253–11.613, *P* = 0.011] and higher CD274⁺ unswitched memory B cells at the end of CPB (adjusted OR=4.516, 95% CI: 1.630–19.508, *P* = 0.002) remained statistically associated with MAEs.

**Conclusion:**

In this single-center study, CPB was associated with dynamic perioperative immune changes, and several immune phenotypes were statistically associated with postoperative MAEs, warranting further validation in larger multicenter studies.

## 1 Introduction

Cardiopulmonary bypass (CPB) is an integral part of cardiac surgery to treat advanced cardiovascular disease, but CPB has been associated with an increased risk of adverse postoperative outcomes and with systemic inflammatory responses involving complement/coagulation activation, ischemia–reperfusion injury, endothelial damage, cytokine release, leukocyte activation, and immune dysregulation (1–4). CPB appears to be accompanied by changes in numerous immune cell populations, which may be associated with adverse postoperative events. For example, previous studies have reported monocyte HLA-DR downregulation and neutrophil CD11b upregulation after CPB, which have been associated with postoperative infection and acute kidney injury, respectively (1, 5). Peripheral blood mononuclear cells can become hyperresponsive to interleukin-6, relative proportions of monocyte subpopulations can change, and interleukin-6 can upregulate CCR2—and many of these changes can persist for weeks after cardiac surgery and have been associated with persistent vascular inflammation (6, 7). Previous studies have also suggested that CPB may be associated with epigenetic and metabolic changes in monocytes and altered cytokine production (8).

We hypothesized that CPB-associated inflammation would be accompanied by perioperative alterations in circulating immune-cell activity and that these alterations might be associated with early postoperative organ injury. Indeed, a higher neutrophil-to-lymphocyte ratio (NLR) (9), which reflects the inflammatory response, is associated with a higher risk of perioperative complications in cardiac surgery (7, 10). To explore our hypothesis further, we examined the numbers and phenotypes of monocytes, neutrophils, natural killer (NK) cells, T cells, and B cells before, during, and after elective CPB cardiac surgery. We explored whether the number and phenotypes were associated with the risk of major adverse postoperative events (MAEs) within 30 days of surgery.

## 2 Materials and methods

### 2.1 Patients

We prospectively enrolled patients older than 18 years who underwent CPB as part of planned valvular surgery and/or coronary artery bypass grafting at the West China Hospital of Sichuan University between May 1, 2022, and March 31, 2023. This study was part of the DIMOCS study (11, 12), which was approved by the Biomedical Ethics Review Committee of West China Hospital (approval 2022–262), registered at ClinicalTrials.gov (NCT05400356), and conducted in accordance with the most recent version of the Declaration of Helsinki. The present report was written in accordance with the “Strengthening the Reporting of Observational Studies in Epidemiology” (STROBE) guidelines (13).

### 2.2 Inclusion and exclusion criteria

Patients (aged ≥ 18 years) scheduled for elective valvular surgery, coronary artery bypass grafting (CABG), or both, requiring CPB were eligible for enrollment. Patients were excluded if they met any of the following criteria: (1) pregnancy; (2) diagnosis of an autoimmune disease, malignancy, hematological disorder, neurological or psychiatric disorder, or chronic inflammatory condition; (3) receipt of immunosuppressive therapy (including monoclonal or polyclonal antibodies, antimetabolites, microbial metabolites, alkylating agents, or radiotherapy) within the preceding 6 months; (4) history of organ or bone marrow transplantation; (5) presence of an active infection, elevated preoperative C-reactive protein levels, or leukocytosis; (6) preoperative renal insufficiency defined as serum creatinine > 176 μmol/L or the requirement for renal replacement therapy; (7) participation in another interventional clinical trial within 30 days prior to surgery or during the current hospitalization; or (8) inability or unwillingness to provide informed consent.

Patients were excluded from the final analysis if the surgery was incomplete, CPB was repeated during the procedure, or consent was withdrawn. Patients who received intraoperative or postoperative blood products were excluded from the final analysis.

### 2.3 Anesthesia and CPB

Surgery and management were performed as described previously (12, 14), and anesthesia was administered according to routine practice at our hospital. Anesthesia was induced with sufentanyl, midazolam, propofol, and vecuronium. Anesthesia was maintained during the bypass with sevoflurane, intermittent sufentanyl, and vecuronium for neuromuscular blockade. Continuous propofol was administered whenever necessary.

Cardiopulmonary bypass was performed according to our institutional routine, as previously described (12, 14). After systemic heparinization (400 IU/kg), CPB was initiated once the activated clotting time exceeded 480 s. The bypass circuit was primed with crystalloid/colloid solution supplemented with mannitol (0.5 g/kg), heparin, and 8.4% sodium bicarbonate (20 mL). Membrane oxygenators were used for gas exchange. Pump flow was maintained according to a target cardiac index of 2.4–2.8 L/min/m^2^, and mean systemic perfusion pressure was maintained between 55 and 75 mmHg. Moderate hypothermia (32–34 °C) was induced at the start of CPB and monitored with a nasopharyngeal temperature probe. Myocardial protection was achieved using intermittent crystalloid cardioplegia with blood cardioplegia at a 4:1 ratio, delivered in an antegrade or retrograde manner according to the surgeon's preference. Hemofiltration was not performed. At the end of CPB, heparin was neutralized with protamine sulfate at a 1:1 ratio. Patients were weaned from CPB when hemodynamically appropriate, with volume support and vasoactive drugs administered as needed. CPB duration and aortic cross-clamp time were recorded for all patients. After surgery, all patients were transferred to the intensive care unit and managed according to standard institutional postoperative protocols.

### 2.4 Blood sampling

Central or peripheral venous blood samples (8 mL) were collected at four perioperative time points: (1) before surgery, (2) rewarming, (3) end of CPB, and (4) 24 h after surgery. During CPB, blood samples were obtained from the venous line of the extracorporeal circuit. Samples were immediately transferred into EDTA-anticoagulated tubes, kept on ice, and analyzed by flow cytometry (see Section 2.4) within 4 h of collection.

### 2.5 Flow cytometry

Multicolor flow cytometry was performed to characterize the abundance and phenotypes of circulating monocytes, T cells (including CD4⁺ and CD8⁺ subsets), B lymphocytes, natural killer (NK) cells, and granulocytes/neutrophils, as previously described, with additional methodological details provided here (12, 15). Peripheral or central venous blood samples (8 mL; Section 2.3) were processed using an in-house red blood cell lysis buffer [8.02 g NH₄Cl, 0.84 g NaHCO₃, and 0.37 g Na₂EDTA·2H₂O dissolved in 1 L phosphate-buffered saline (PBS)]. Aliquots of the leukocyte suspension (100 μL) were distributed into separate tubes and incubated for 30 min at room temperature in the dark with fluorophore-conjugated antibodies against immune cell surface markers. An unstained control was included in each experiment. After staining, samples were centrifuged at 300 × g for 5 min at 4 °C, the supernatant was discarded, and the cell pellet was resuspended in 300 μL phosphate-buffered saline (PBS).

Separate antibody panels were designed for T cells, B cells, monocytes, NK cells, and granulocytes/neutrophils (Supplementary Table S1). In each panel, lineage markers were used to identify the target population, whereas functional markers were used to assess activation, co-stimulatory/co-inhibitory signaling, and effector-related phenotypes. Briefly, T cells were identified using CD45, CD3, CD4, and CD8, with CD45RO used for memory/effector phenotyping, CD69 and CD38 for activation, CD28 for co-stimulation, and CD279 (PD-1) for checkpoint-related inhibitory/exhaustion phenotypes. B cells were identified using CD45 and CD19 and further classified by IgD and CD27 expression, with additional markers [IgM, IgG, CD38, CD80, and CD274 (PD-L1)] used for phenotypic characterization. NK cells were defined as CD3⁻ lymphocytes within CD45⁺ leukocytes and further characterized by CD16, CD56, CD57, CD335 (NKp46), CD314 (NKG2D), and CD127. Neutrophils were identified based on CD45 expression and CD14/CD15 expression, with CD54, CD11b, CD181, CD64, CD123, and Siglec-8 used for phenotypic analysis. Monocyte subsets were defined by CD14/CD16 expression, with HLA-DR, CD80, CD40, CD163, CD274 (PD-L1), and CD284 (TLR4) used to assess inflammatory and immunoregulatory phenotypes.

Samples were acquired on a FACSLyric™ flow cytometer (BD Biosciences, Franklin Lakes, NJ, USA). Daily instrument quality control was performed using the manufacturer's program, and acquisition was performed only after passing quality control. Single-stain controls were used for fluorescence compensation, and acquisition settings were kept consistent within each batch. Data were analyzed using FlowJo software (Tree Star, Ashland, OR, USA). The operator was blinded to sample identity and clinical outcomes.

The gating strategy followed a standardized sequential approach (Supplementary Figure S1). Briefly, acquisition stability was assessed using Time and SSC-A parameters. Debris, doublets, and dead cells were excluded using FSC/SSC characteristics, FSC-A versus FSC-H gating, and viability dye staining. Major leukocyte populations were then identified based on CD45 expression and lineage-specific markers, followed by sequential gating of immune subpopulations according to marker expression. A standardized gating template was applied across all samples to ensure comparability.

### 2.6 Data collection and follow-up

All patients were assessed by trained research nurses within 24 h of admission. Demographic data, clinical characteristics, medical history, and laboratory data were also collected. Perioperative data were obtained from the medical records. Patients were followed up for 30 days after surgery, whether in the hospital or after discharge. Follow-up data were collected via telephone calls using structured questionnaires or hospital records.

The primary outcome was major adverse postoperative events (MAEs) occurring within 30 days after surgery. This prespecified composite endpoint was intended to capture early clinically significant postoperative multi-organ dysfunction after CPB and included acute kidney injury, neurological dysfunction, liver injury, cardiovascular complications, and respiratory dysfunction. These components were selected *a priori* because they represent major postoperative complications across organ systems and are clinically relevant to CPB-related systemic inflammation, ischemia–reperfusion injury, endothelial dysfunction, and perioperative immune dysregulation (16, 17). Definitions of individual endpoint components were based on established consensus criteria (18) and are provided in the Supplementary Definitions.

### 2.7 Statistics

Data were analyzed statistically using R 4.1.0 (R Foundation for Statistical Computing, Vienna, Austria). Continuous data were examined for normality using the Shapiro–Wilk test and presented as mean ± standard deviation if normally distributed, or as median [interquartile range (IQR)] if skewed. Categorical data were summarized as frequencies and percentages. Between-group comparisons were conducted using the independent-samples *t* test or Mann–Whitney *U* test for continuous variables, and the *χ*^2^ test or Fisher's exact test, as appropriate, for categorical variables.

Where appropriate, data distributions were displayed as box-and-whisker plots generated with the *ggplot2* package in R, with boxes indicating the 25th, 50th, and 75th percentiles and whiskers representing the minimum and maximum.

For repeated measurements across perioperative time points within the same individuals, pairwise comparisons were performed using the paired Wilcoxon signed-rank test (matched by subject ID). Bonferroni correction was applied for the six pairwise comparisons within each variable, and only comparisons that remained statistically significant after correction (adjusted *P* < 0.05) were annotated in the figures.

Univariate logistic regression was performed to assess the association between clinical variables and postoperative outcomes. Receiver operating characteristic curves (ROC) were generated using the pROC package in R to estimate the area under the curve (AUC) and corresponding confidence intervals for distinguishing patients with and without MAEs. Given the limited number of MAEs, these analyses were not intended to evaluate predictive performance or to define clinically applicable cutoffs.

Given the limited number of MAEs, we used Firth penalized logistic regression for risk-adjusted analyses to reduce small-sample bias. To minimize overfitting and multiple testing, multivariable models were restricted *a priori* to four immune subsets central to our study hypothesis (CD163^+^ classical monocytes and CD284^+^ non-classical monocytes at rewarming; CD274^+^ unswitched memory B cells and CD4^+^ CD38^+^ T cells at the end of CPB) and adjusted for age, preoperative eGFR, and preoperative CRP; a sensitivity analysis additionally adjusted for preoperative AST. To further assess potential perioperative confounding, additional sensitivity analyses were performed by incorporating cardiopulmonary bypass duration and aortic cross-clamp time into the parsimonious models one at a time. Because surgical procedures were not mutually exclusive and some categories were sparse, individual procedure types were not included in the main models to avoid overfitting and collinearity.

All statistical tests were two-sided, and results were considered statistically significant at *P* < 0.05. Given the large number of immune phenotypes, subgroup comparisons, and repeated time-point analyses, the risk of type I error cannot be excluded. Therefore, all unadjusted between-group comparisons and ROC analyses should be considered exploratory screening analyses. Apart from Bonferroni correction applied to predefined repeated-measures comparisons across time points, no global adjustment for multiple testing was performed. Consequently, statistically significant findings should be interpreted as hypothesis-generating rather than confirmatory.

## 3 Results

### 3.1 Patient characteristics

**Figure 1:**
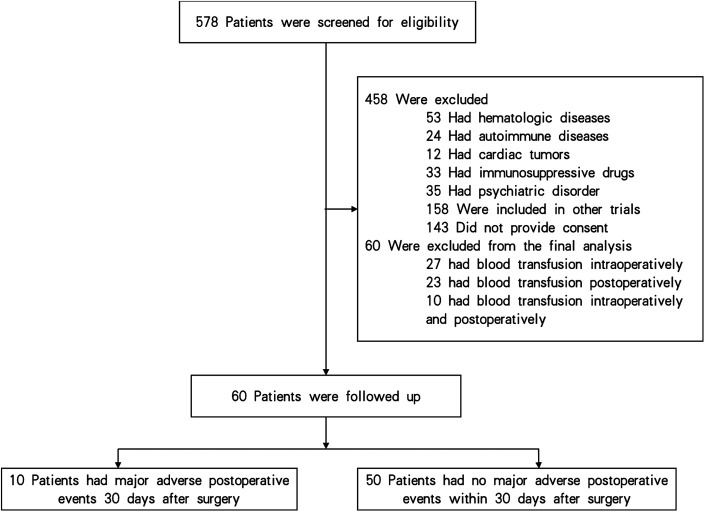
Flowchart of patient enrollment and analysis.

A total of 578 patients were screened for eligibility, and 60 (29 men) met the inclusion criteria and were ultimately included in the final analysis (Figure 1). The mean patient age was 56 ± 8 years. Among the 60 patients, 10 (16.7%) experienced MAEs within 30 days after surgery, including acute kidney injury (*n* = 4, 6.7%), neurological dysfunction (*n* = 3, 5.0%), and liver injury (*n* = 3, 5.0%) (Table 1). No cardiovascular or respiratory events were observed in this cohort, and no patient experienced more than one component of MAEs. Compared with patients without MAEs, those who experienced MAEs were significantly older (*P* = 0.035) and had higher serum creatinine (*P* = 0.012), aspartate aminotransferase (*P* = 0.030), and C-reactive protein (*P* = 0.020) levels (Table 2).

**Table 1:** Frequencies of individual components of major adverse postoperative events (MAEs) within 30 days after surgery.

Outcomes	*n* (%)
MAEs	10 (16.7)
Acute kidney injury	4 (6.7)
Neurological dysfunction	3 (5.0)
Liver injury	3 (5.0)
Cardiovascular complications	0 (0)
Respiratory dysfunction	0 (0)

MAEs, major adverse postoperative events.

**Table 2:** Preoperative and perioperative characteristics stratified by major adverse postoperative events (MAEs).

Characteristic	Major adverse postoperative events (MAEs)		*p*
	No (*n* = 50)	Yes (*n* = 10)	
Demographics			
Age, yr	55.22 ± 8.74	60.30 ± 5.85	0.035
Men	22 (44)	7 (70)	0.200
American Society of Anesthesiologists physical status			0.600
Ⅲ	43 (86)	8 (80)	
Ⅳ	7 (14)	2 (20)	
New York Heart Association classification			0.700
Ⅰ	2 (4.0)	0 (0)	
Ⅱ	30 (60)	5 (50)	
Ⅲ	17 (34)	5 (50)	
Ⅳ	1 (2.0)	0 (0)	
Body mass index, kg/m^2^	24.19 ± 3.45	23.06 ± 2.55	0.200
Smoking history	11 (22)	4 (40)	0.200
Drinking history	9 (18)	3 (30)	0.400
Medical history			
Hypertension	14 (28)	3 (30)	> 0.9
Hyperlipemia	16 (32)	3 (30)	> 0.9
Coronary artery disease	7 (14)	2 (20)	0.600
Arrhythmia	23 (46)	7 (70)	0.300
Diabetes mellitus	3 (6.0)	2 (20)	0.200
Leukocytes, × 10^9^/L	5.96 (5.01, 6.79)	6.32 (6.09, 7.10)	0.200
Neutrophils, × 10^9^/L	3.36 (2.66, 4.30)	3.85 (3.34, 4.45)	0.200
Lymphocytes, × 10^9^/L	1.74 (1.42, 2.16)	2.04 (1.75, 2.46)	0.110
Hemoglobin, g/L	137.92 ± 12.10	147.50 ± 16.18	0.100
Platelets, × 10^9^/L	181.90 ± 55.27	182.20 ± 72.11	> 0.9
Monocytes, × 10^9^/L	0.38 ± 0.13	0.49 ± 0.25	0.200
Red blood cells, × 10^12^/L	4.65 (4.24, 4.92)	4.95 (4.48, 5.07)	0.400
Mean corpuscular volume, fL	92.95 (89.30, 96.20)	93.85 (92.10, 95.30)	0.500
Mean corpuscular			
hemoglobin, pg	30.15 (29.20, 31.10)	30.65 (29.30, 31.30)	0.400
hemoglobin concentration, g/L	323.00 (317.00, 329.00)	328.00 (323.00, 337.00)	0.200
Urea, mmol/L	5.55 (4.90, 6.90)	6.00 (5.00, 8.10)	0.400
Serum creatinine, µmol/L	74.50 (63.00, 84.00)	89.00 (77.00, 99.00)	0.012
Creatinine clearance, mL/min	81.00 (65.00, 96.00)	62.50 (59.00, 73.00)	0.027
eGFR, mL/min/1.73m^2^	89.41 ± 14.35	73.47 ± 15.43	0.010
Aspartate aminotransferase, IU/L	21.00 (18.00, 24.00)	27.50 (21.00, 35.00)	0.030
Alanine aminotransferase, IU/L	19.00 (13.00, 25.00)	19.50 (13.00, 46.00)	0.400
Total bilirubin, μmol/L	14.35 (11.40, 17.70)	14.55 (10.20, 27.90)	0.500
Albumin, g/L	44.24 ± 2.85	46.18 ± 4.09	0.200
Total cholesterol, mmol/L	4.66 ± 0.97	4.73 ± 1.09	0.900
HDL-cholesterol, mmol/L	1.41 ± 0.37	1.38 ± 0.31	0.800
LDL-cholesterol, mmol/L	2.81 ± 0.83	2.85 ± 0.85	> 0.9
Triglycerides, mmol/L	1.14 (0.95, 1.59)	1.33 (1.15, 1.52)	0.200
Glucose, mmol/L	5.23 (4.76, 5.70)	5.16 (4.71, 6.06)	0.800
C-reactive protein, mg/L	1.40 (0.00, 2.70)	2.79 (1.77, 7.98)	0.020
Prothrombin time, s	11.05 (10.50, 12.10)	11.20 (10.60, 14.40)	0.700
International normalized ratio	1.04 (0.97, 1.12)	1.02 (0.98, 1.29)	0.800
APTT, s	27.10 (26.00, 29.50)	29.00 (26.90, 30.60)	0.100
Fibrinogen, g/L	2.65 ± 0.72	3.01 ± 0.59	0.110
Cardiac troponin T, ng/L	9.30 (7.00, 12.00)	10.85 (9.30, 25.80)	0.200
LVEF (%), median [IQR]	64.00 (58.00, 69.00)	66.00 (60.00, 69.00)	0.600
CPB duration, min	126.06 ± 38.82	147.20 ± 50.12	0.200
Total urine output during CPB, mL	542.50 (320.00, 985.00)	420.00 (340.00, 680.00)	0.500
Aortic cross-clamping time, min	85.50 (67.00, 104.00)	109.50 (85.00, 128.00)	0.058
Surgical time, h	4.19 ± 0.87	4.60 ± 0.95	0.200
CABG	3 (6)	0 (0)	> 0.9
Valve replacement	49 (98)	10 (100)	0.700
Aortic surgery	7 (14)	1 (10)	> 0.9
Radiofrequency ablation	15 (30)	5 (50)	0.300
Length of ICU stay, day	2.46 (1.84, 3.84)	2.48 (2.01, 3.80)	0.400
Length of postoperative hospitalization, day	7.00 (7.00, 9.00)	7.00 (7.00, 8.00)	0.600
Postoperative intubation time, h	17.19 (9.33, 19.92)	12.15 (9.13, 20.82)	> 0.9

Data are reported as *n* (%), mean ± SD or median (interquartile range) unless otherwise noted. APTT, activated partial thromboplastin time; CABG, coronary artery bypass grafting; CPB, cardiopulmonary bypass; eGFR, estimated glomerular filtration rate; ICU, intensive care unit; SD, standard deviation.

### 3.2 Perioperative dynamics of leukocyte counts and inflammatory indices

**Figure 2:**
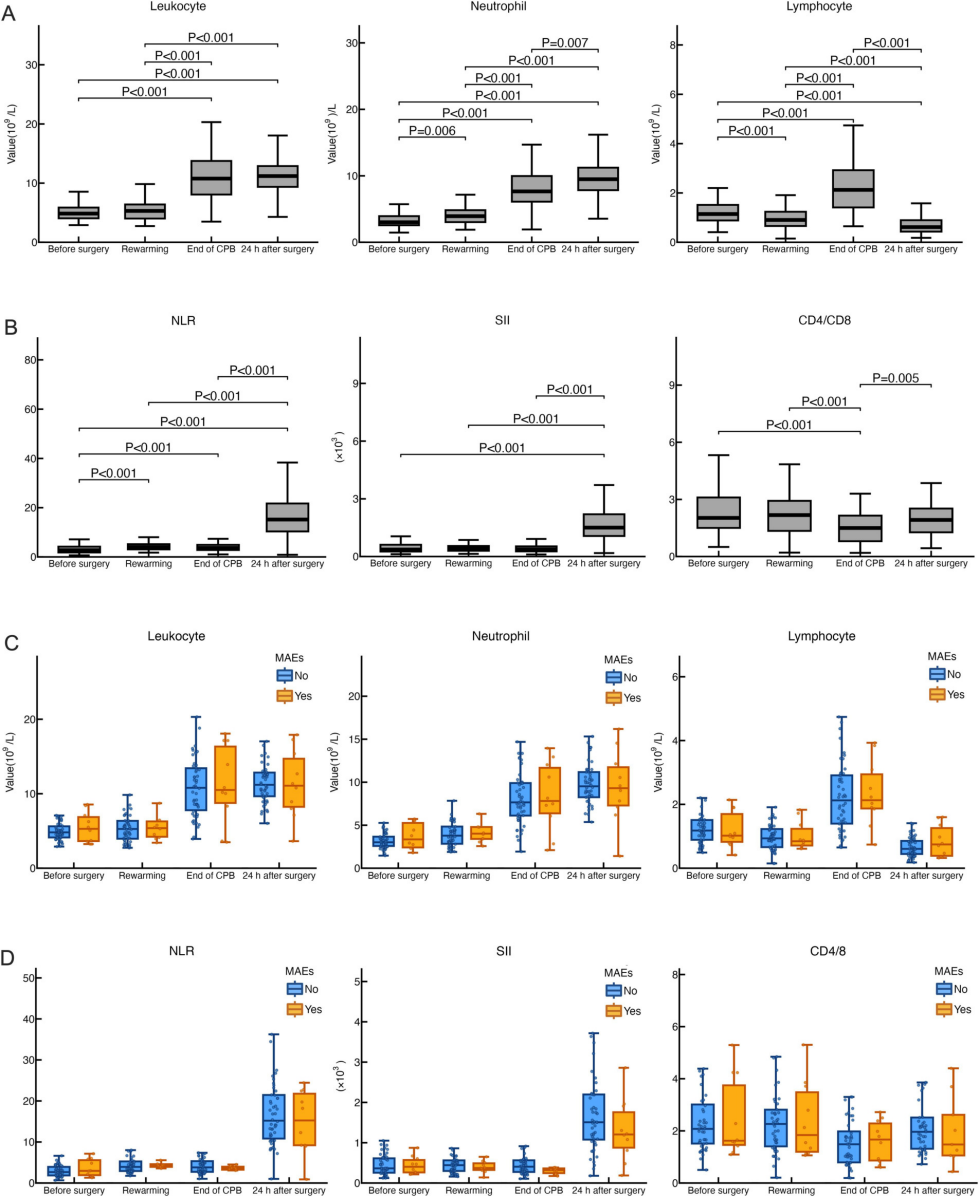
**(A,B)** Trajectories of the absolute counts of the indicated populations of blood cells, the neutrophil-to-lymphocyte ratio (NLR), systemic immune-inflammation index (SII), and ratio of CD4^+^ to CD8^+^ T cells at different times before, during and after cardiopulmonary bypass (CPB). **(C,D)** Differences in the results between the 10 patients who experienced major adverse postoperative events (MAEs) within 30 days after surgery and the 50 patients who did not. CD4/8, the ratio of CD4^+^ to CD8^+^ T cells.

CPB was associated with marked perioperative changes in leukocyte counts. Total leukocyte count increased from 5.19 ± 1.68 × 10⁹/L before surgery to 10.84 ± 4.07 × 10⁹/L at the end of CPB, peaking at 11.41 ± 3.27 × 10⁹/L at 24 h after surgery. The perioperative trajectories of neutrophils and lymphocytes differed markedly. Absolute neutrophil count rose continuously throughout the study period, increasing from 3.37 ± 1.23 × 10⁹/L before surgery to 4.19 ± 1.83 × 10⁹/L during rewarming, 8.20 ± 3.23 × 10⁹/L at the end of CPB, and 9.61 ± 3.15 × 10⁹/L at 24 h after surgery. In contrast, absolute lymphocyte count decreased during rewarming from 1.29 ± 0.67 × 10⁹/L to 1.02 ± 0.56 × 10⁹/L, showed a transient rise at the end of CPB to 2.23 ± 1.05 × 10⁹/L, and subsequently fell to 0.69 ± 0.34 × 10⁹/L at 24 h after surgery (Figure 2A).

Inflammatory indices also changed substantially over time. The neutrophil-to-lymphocyte ratio (NLR) increased from 3.06 ± 1.48 before surgery to 17.03 ± 8.84 at 24 h after surgery, while the systemic immune-inflammation index (SII) (19) rose from 0.45 ± 0.24 × 10^3^ to 1.83 ± 1.35 × 10^3^ over the same period. In parallel, the CD4⁺/CD8⁺ ratio declined from 2.42 ± 1.33 before surgery to 1.58 ± 0.93 at the end of CPB and then increased to 2.25 ± 1.33 at 24 h after surgery (Figure 2B).

None of the leukocyte counts, ratios, or indices differed significantly at any of the four time points between patients who did or did not experience MAEs (Figures 2C,D).

### 3.3 Perioperative dynamics of lymphocytes: NK cells, T cells and B cells

**Figure 3:**
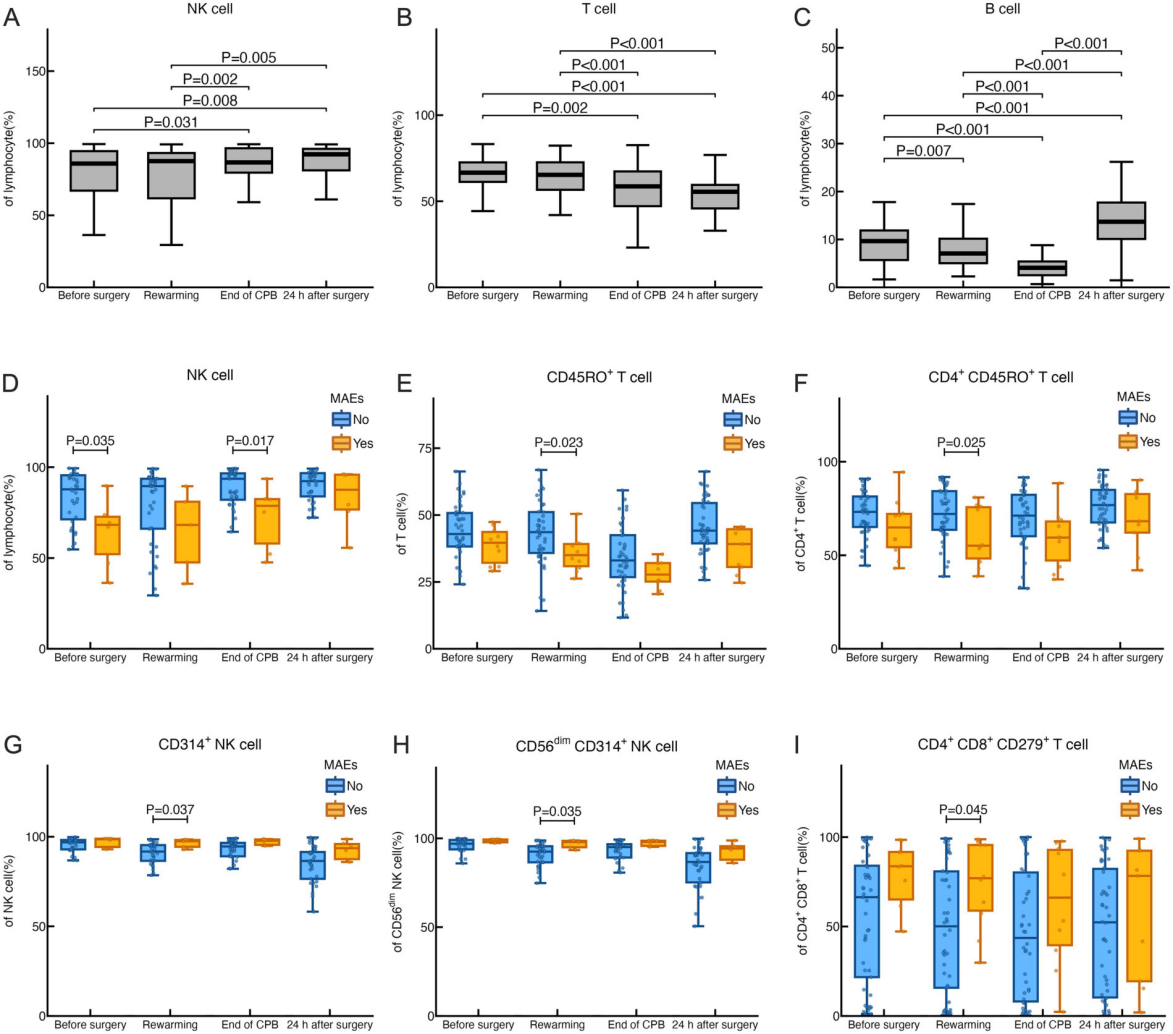
**(A–C)** Trajectories of the relative abundance of natural killer (NK) cells, T and B cells at different times before, during and after cardiopulmonary bypass (CPB). **(D–I)** Comparison of the proportions of total NK cells, total NK cells and their subpopulations between the 10 patients who experienced major adverse postoperative events (MAEs) within 30 days after surgery and the 50 patients who did not.

First, we analyzed the proportions of cell subpopulations across the four time points in the entire cohort. The proportion of NK cells among lymphocytes remained relatively stable from preoperative (79.34 ± 18.18%) to rewarming (76.93 ± 21.03%), then increased at the end of CPB (85.87 ± 13.14%) and again at 24 h after surgery (87.80 ± 11.50%; Figure 3A). The proportion of T cells among lymphocytes remained stable from preoperative (65.21 ± 10.78%) to rewarming (64.40 ± 9.86%), then decreased at the end of CPB (57.28 ± 13.34%) and again 24 h after surgery (53.68 ± 9.96%; Figure 3B). The proportion of B cells among lymphocytes decreased from preoperative (9.12 ± 3.74%) to rewarming (7.68 ± 3.50%), reached a nadir at the end of CPB (4.51 ± 2.53%), and then substantially rebounded 24 h after surgery (13.95 ± 5.73%; Figure 3C).

Next, we compared the trajectories of cell subpopulations between patients who did and did not experience MAEs. Those who experienced MAEs showed significantly lower proportions of NK cells before surgery (63.90 ± 19.23 vs. 81.99 ± 16.89%, *P* = 0.035) and at the end of CPB (72.23 ± 18.32 vs. 88.08 ± 10.90%, *P* = 0.017; Figure 3D). They also exhibited lower proportions of CD45RO⁺ T cells (35.62 ± 6.93 vs. 43.22 ± 11.66%, *P* = 0.023) and CD4⁺ CD45RO⁺ T cells (60.03 ± 15.64 vs. 72.36 ± 13.75%, *P* = 0.025; Figures 3E,F) during rewarming. Conversely, those patients during rewarming showed significantly higher proportions of CD314⁺ NK cells (96.30 ± 2.50 vs. 88.52 ± 11.05%, *P* = 0.037) and CD56^dim^ CD314⁺ NK cells (96.74 ± 2.41 vs. 88.41 ± 11.75%, *P* = 0.035) (Figures 3G,H), as well as CD4⁺ CD8⁺ CD279⁺ T cells (73.40 ± 24.68 vs. 48.52 ± 35.88%, *P* = 0.045; Figure 3I).

**Figure 4:**
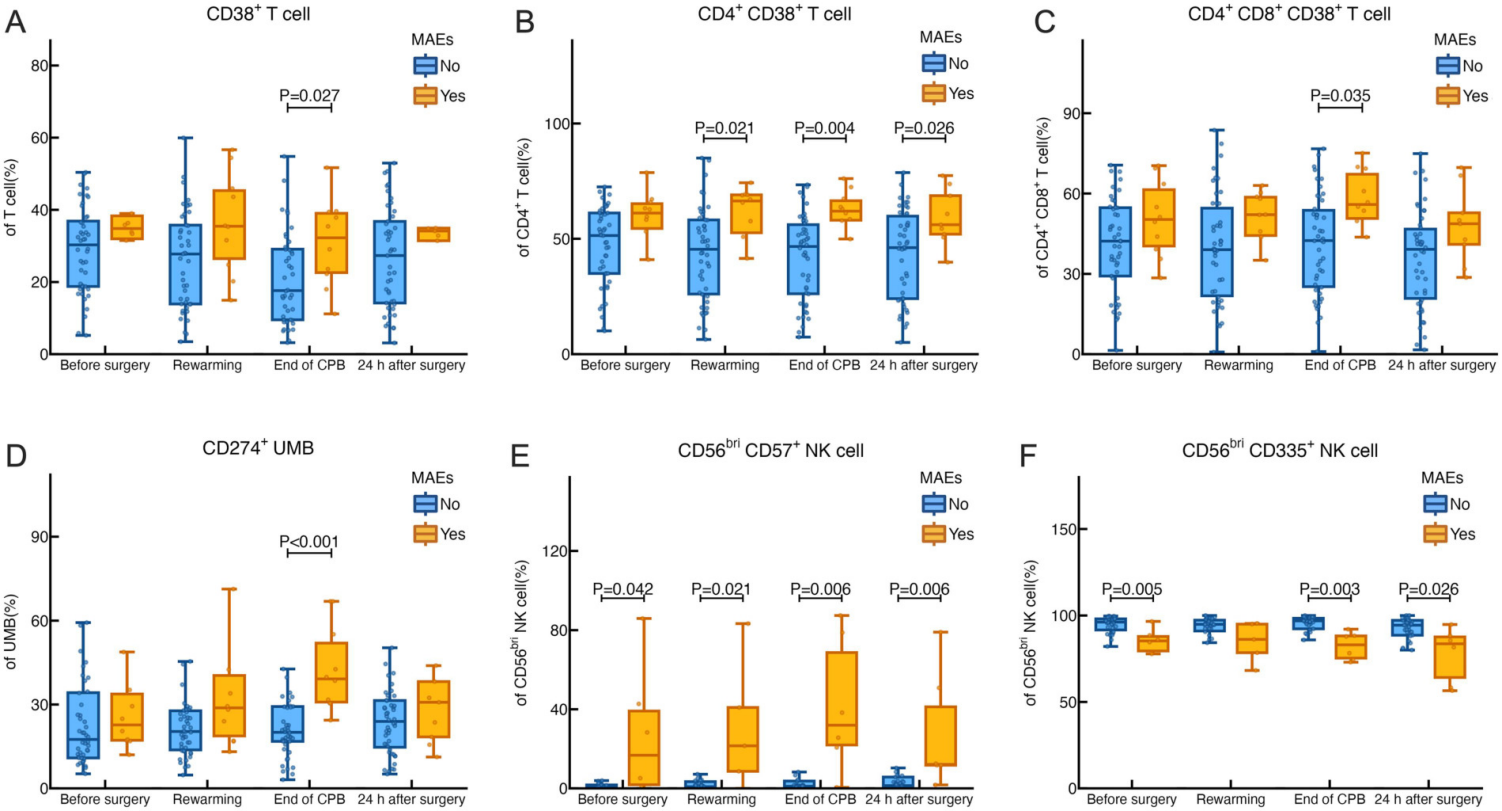
**(A–F)** Comparison of the proportions of T cell subpopulations, B cell subpopulations and natural killer (NK) cell subpopulations between the 10 patients who experienced major adverse postoperative events (MAEs) within 30 days after surgery and the 50 patients who did not. UMB, unswitched memory B cells.

At the end of CPB, patients who experienced MAEs showed significantly higher proportions of CD38⁺ T cells (30.80 ± 12.18 vs. 20.95 ± 12.49%, *P* = 0.027), CD4⁺CD38⁺ T cells (59.16 ± 15.16 vs. 41.78 ± 18.60%, *P* *=* 0.004), and CD4⁺ CD8⁺ CD38⁺ T cells (54.48 ± 18.19 vs. 40.95 ± 18.68%, *P* = 0.035; Figures 4A–C) as well as CD274⁺ unswitched memory B cells (42.69 ± 15.29 vs. 23.80 ± 15.04%, *P* < 0.001; Figure 4D).

The perioperative proportions of CD4⁺CD38⁺ T cells were persistently higher among patients who experienced MAEs, whether at rewarming (58.67 ± 16.25 vs. 43.65 ± 20.19%, *P* = 0.021), the end of CPB (59.16 ± 15.16 vs. 41.78 ± 18.60%, *P* = 0.004), or 24 h after surgery (59.31 ± 12.07 vs. 43.16 ± 19.42%, *P* = 0.026; Figure 4B). Those patients also showed higher proportions of CD56^bri^ CD57⁺ NK cells, whether before surgery (27.13 ± 33.51 vs. 2.99 ± 9.29%, *P* = 0.042), during rewarming (30.86 ± 33.11 vs. 2.83 ± 5.01%, *P* = 0.021), at the end of CPB (41.88 ± 34.28 vs. 5.51 ± 12.97%, *P* = 0.006) or at 24 h after surgery (27.91 ± 30.27 vs. 6.04 ± 11.59%, *P* = 0.006; Figure 4E). They showed lower proportions of CD56^bri^ CD335⁺ NK cells at preoperative (82.50 ± 11.95 vs. 93.43 ± 9.85%, *P* = 0.005), at the end of CPB (82.32 ± 8.11 vs. 92.71 ± 12.02%, *P* = 0.003), and 24 h after surgery (77.53 ± 16.18 vs. 89.52 ± 14.29%, *P* = 0.026; Figure 4F).

Patients who experienced MAEs or not did not differ from each other significantly in the proportions of CD57⁺, CD56^bri^, CD56^dim^, or CD335⁺ NK cells at four time points (Supplementary Figures S2A–D). There were no differences in the proportions of total, CD4⁺, CD4⁺ CD8⁺ or CD279⁺ T cells at the four time points (Supplementary Figures S2E–H), nor did they differ in the proportions of total B cells, unswitched memory B cells, or CD274⁺ B cells at the four time points (Supplementary Figures S2I–K).

**Figure 5:**
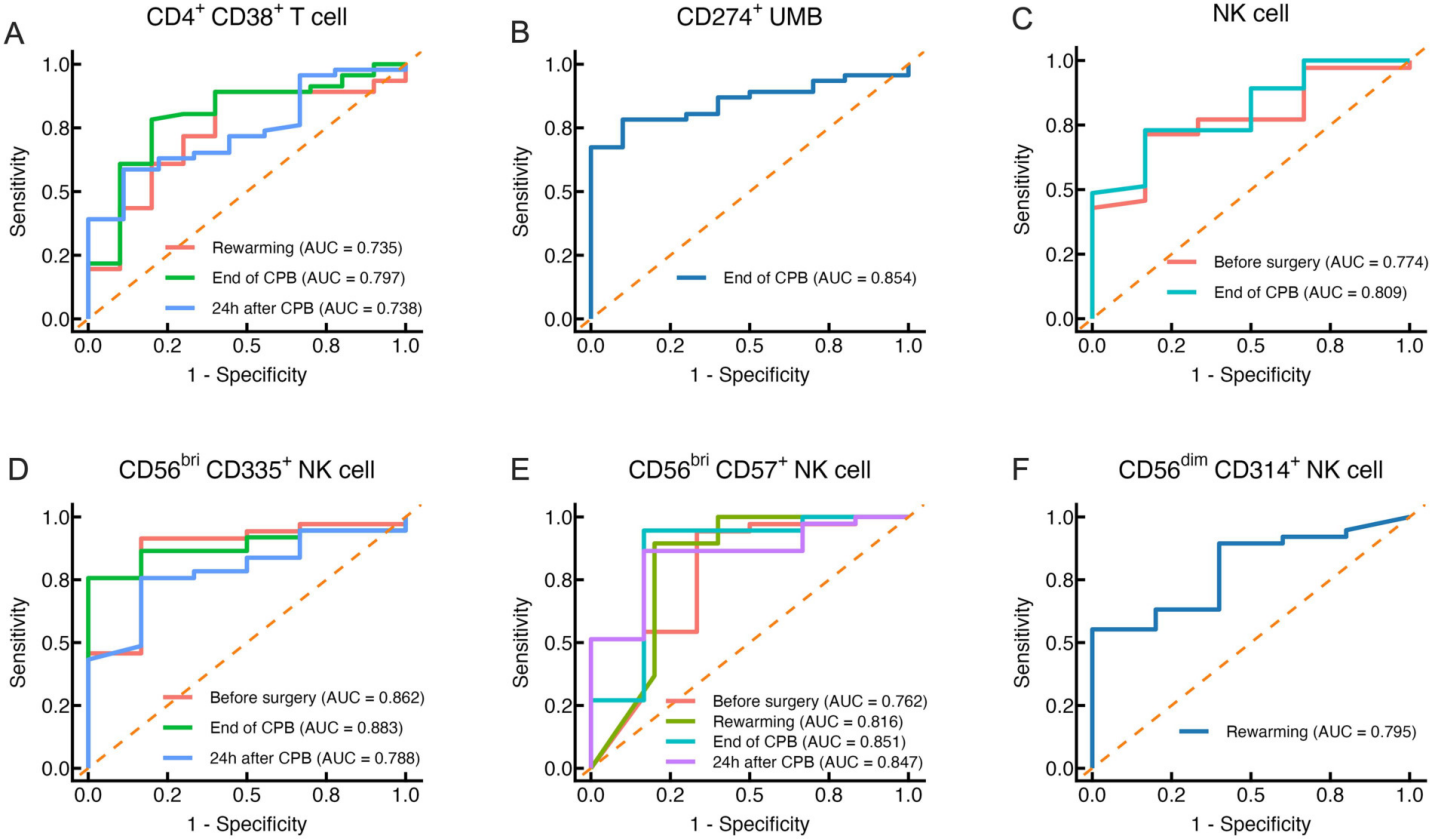
**(A–F)** Exploratory receiver operating characteristic curves (ROC) describing within-cohort discrimination between patients with and without major adverse postoperative events (MAEs) based on the proportions of CD4⁺CD38⁺ T cells, CD274⁺ unswitched memory B cells, total natural killer (NK) cells, and selected NK-cell subpopulations. Areas under the curves (AUCs) are shown at the lower right. UMB, unswitched memory B cells.

Finally, exploratory ROC analyses were performed to describe the within-cohort separation of selected immune cell subpopulations between patients with and without MAEs. The proportion of CD4⁺CD38⁺ T cells showed AUC values of 0.735 at rewarming, 0.797 at the end of CPB, and 0.738 at 24 h after surgery (Figure 5A). The proportion of CD274⁺ unswitched memory B cells showed an AUC of 0.854 at the end of CPB (Figure 5B). The proportions of total NK cells, CD56briCD335⁺ NK cells, CD56briCD57⁺ NK cells, CD56dimCD314⁺ NK cells, and CD314⁺ NK cells showed AUC values exceeding 0.790 at multiple time points (Figures 5C–F; Supplementary Figure S3F). Other immune phenotypes, including CD45RO⁺ T cells, CD4⁺CD45RO⁺ T cells, CD4⁺CD8⁺CD279⁺ T cells, CD38⁺ T cells, and CD4⁺CD8⁺CD38⁺ T cells, showed AUC values ranging from 0.704 to 0.733 (Supplementary Figures S3A–E).

### 3.4 Perioperative dynamics of monocytes and neutrophils

**Figure 6:**
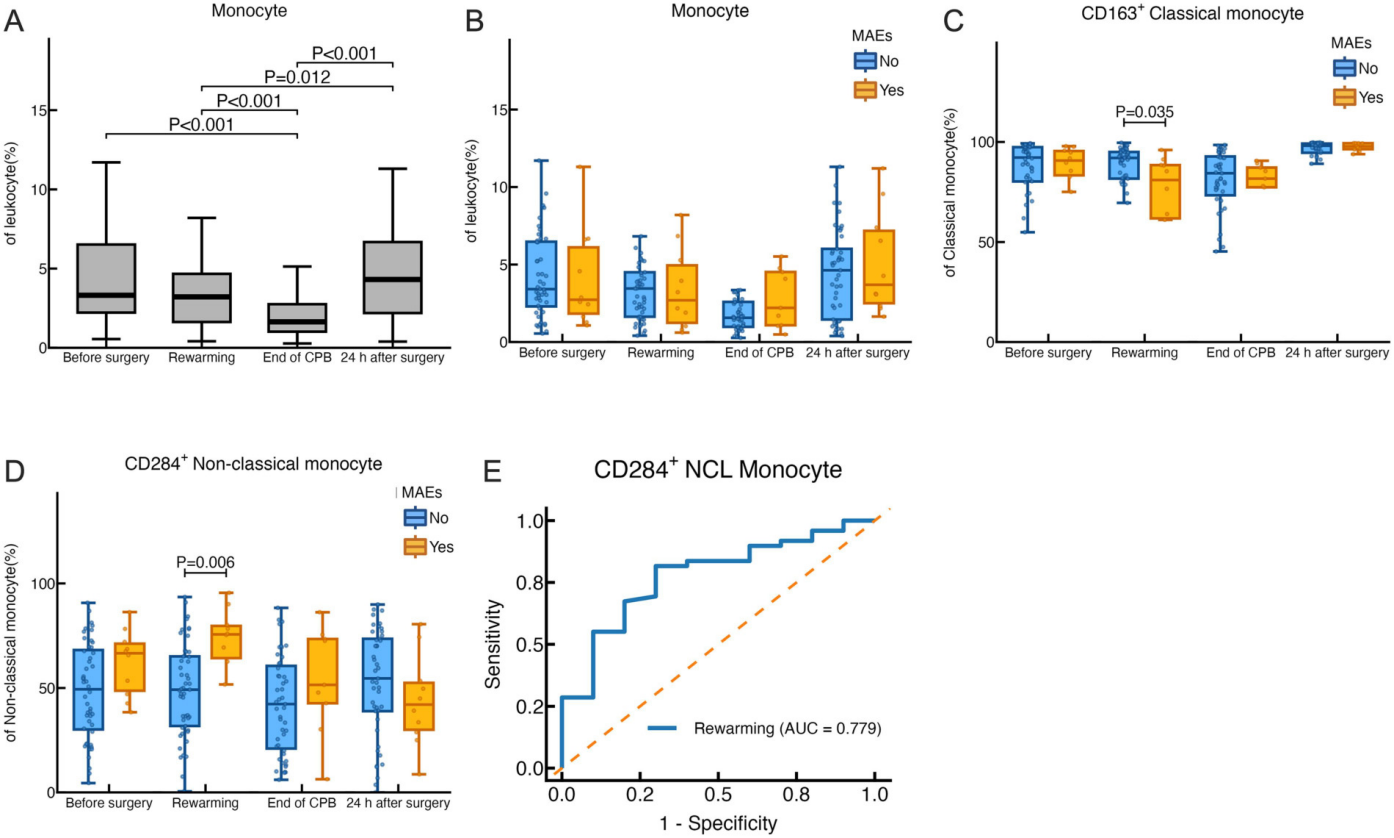
**(A)** Trajectory of the relative abundance of monocytes at different times before, during and after cardiopulmonary bypass (CPB). **(B–D)** Comparison of the proportions of monocytes and their subpopulations between the 10 patients who experienced major adverse postoperative events (MAEs) within 30 days after surgery and the 50 patients who did not. **(E)** Exploratory receiver operating characteristic curve (ROC) describing within-cohort discrimination between patients with and without major adverse postoperative events (MAEs) based on the proportion of CD284⁺ non-classical monocytes at rewarming. The area under the curve (AUC) is shown at the lower right. NCL, non-classical monocyte.

First, we analyzed the proportions of cell subpopulations across the four time points in the entire cohort. The proportion of monocytes among leukocytes gradually decreased from preoperative (4.23 ± 2.78%) to rewarming (3.59 ± 2.69%) and reached a nadir at the end of CPB (2.12 ± 1.55%), followed by a rebound to levels close to preoperative, 24 h after surgery (4.56 ± 2.95%; Figure 6A). The proportion of neutrophils among granulocytes progressively increased from preoperative (93.73 ± 6.12%) to rewarming (96.64 ± 2.24%) and the end of CPB (98.39 ± 1.01%) and remained elevated 24 h after surgery (98.26 ± 1.82%; Supplementary Figure S5A).

Next, we compared the trajectories of monocyte and neutrophil subpopulations between patients who did and did not experience MAEs. Patients who experienced MAEs had a significantly lower proportion of CD163⁺ classical monocytes during rewarming (69.46 ± 27.59 vs. 85.04 ± 15.53%, *P* = 0.035) and a higher proportion of CD284⁺ non-classical monocytes (71.36 ± 17.95 vs. 49.13 ± 22.42%, *P* = 0.006) during rewarming (Figures 6C,D).

The overall proportion of monocytes was comparable between the two groups throughout the perioperative period (Figure 6B), and no significant between-group differences were observed in the proportions of classical and non-classical monocytes (Supplementary Figures S4A,B). Likewise, no significant between-group differences were observed across the four time points in the proportions of CD163⁺ monocytes or CD284⁺ monocytes (Supplementary Figures S4C,D). The same was observed for the proportions of total, classical, and non-classical HLA-DR⁺ monocytes (Supplementary Figures S4E–G) and for the proportions of total, CD11b⁺, CD54⁺, CD181⁺, and CD64⁺ neutrophils (Supplementary Figures S5B–F).

In exploratory ROC analyses, the proportion of CD163⁺ classical monocytes showed an AUC of 0.714 (Supplementary Figure S4H), whereas the proportion of CD284⁺ non-classical monocytes showed an AUC of 0.779 for separating patients with and without MAEs within this cohort (Figure 6E).

### 3.5 Risk-adjusted and sensitivity analyses of pre-specified immune markers

To further assess whether the observed associations were robust after accounting for preoperative differences and the limited number of events, we performed Firth penalized logistic regression for four pre-specified immune markers (per 1-SD increase), adjusting for age, preoperative estimated glomerular filtration rate (eGFR), and preoperative C-reactive protein (CRP) (Table 3). Higher proportions of CD284⁺ non-classical monocytes at rewarming (adjusted OR 3.118, 95% CI 1.253–11.613; *P* = 0.011) and CD274⁺ unswitched memory B cells at the end of CPB (adjusted OR 4.516, 95% CI 1.630–19.508; *P* = 0.002) remained statistically associated with MAEs. CD4⁺CD38⁺ T cells at the end of CPB showed a borderline association (adjusted OR 2.480, 95% CI 1.000–8.178; *P* = 0.050), whereas CD163⁺ classical monocytes at rewarming were not significantly associated with MAEs after adjustment (adjusted OR 0.518, 95% CI 0.210–1.195; *P* = 0.126).

**Table 3:** Associations between perioperative immune markers and major adverse postoperative events (MAEs) using Firth penalized logistic regression.

Immune marker (per 1-SD increase)	Adjusted OR (95% CI)	*P* value	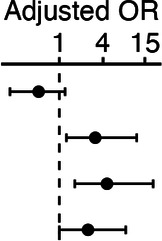

CD163⁺ classical monocytes (Rewarming)	0.52 (0.21–1.20)	0.126	
CD284⁺ non-classical monocytes (Rewarming)	3.12 (1.25–11.61)	0.011	
CD274⁺ unswitched memory B cells (End of CPB)	4.52 (1.63–19.51)	0.002	
CD4⁺CD38⁺ T cells (End of CPB)	2.48 (1.00–8.18)	0.050	

Models were adjusted for age, preoperative estimated glomerular filtration rate (eGFR), preoperative C-reactive protein (CRP). Immune markers were z-score standardized (per 1-SD increase); ORs therefore represent the change in odds of MAEs per 1-SD increase in marker proportion. Firth penalized logistic regression was applied given the limited number of MAEs. To minimize overfitting given the low event rate, multivariable analyses were restricted *a priori* to four immune subsets central to the study hypothesis.

In sensitivity analyses additionally adjusting for preoperative aspartate aminotransferase (AST), the overall pattern of results was broadly similar (Supplementary Table S2). CD284⁺ non-classical monocytes at rewarming (adjusted OR 2.54, 95% CI 1.03–9.62; *P* = 0.042) and CD274⁺ unswitched memory B cells at the end of CPB (adjusted OR 3.64, 95% CI 1.36–16.38; *P* = 0.008) remained statistically associated with MAEs, whereas the association for CD4⁺CD38⁺ T cells was attenuated.

Additional sensitivity analyses were performed to account for perioperative exposure variables by adding CPB duration or aortic cross-clamp time to the base model (Table 4). Under these perioperative-adjusted models, the associations for the key markers remained directionally consistent. CD284⁺ non-classical monocytes remained significantly associated with MAEs after adjustment for CPB duration (OR 2.50, 95% CI 1.01–8.90; *P* = 0.047), but the association was attenuated after adjustment for aortic cross-clamp time (OR 2.17, 95% CI 0.88–7.72; *P* = 0.097). In contrast, CD274⁺ unswitched memory B cells remained significantly associated with MAEs in both perioperative-adjusted models (*P* = 0.006 and *P* = 0.012, respectively).

**Table 4:** Sensitivity analyses additionally adjusting for perioperative variables (CPB time and aortic cross-clamp time) in Firth penalized logistic regression models for key immune markers.

Model 3: Base model (Age, eGFR, CRP) + CPB duration		
Immune marker (per 1-SD increase)	Adjusted OR (95% CI)	*P* value
CD284⁺ non-classical monocytes (Rewarming)	2.50 (1.01–8.90)	0.047
CD274⁺ unswitched memory B cells (End of CPB)	4.07 (1.45–18.74)	0.006
Model 4: Base model (Age, eGFR, CRP) + Aortic cross-clamp time		
Immune marker (per 1-SD increase)	Adjusted OR (95% CI)	*P* value
CD284⁺ non-classical monocytes (Rewarming)	2.17 (0.88–7.72)	0.097
CD274⁺ unswitched memory B cells (End of CPB)	3.86 (1.34–18.10)	0.012

Models were fitted using Firth penalized logistic regression. Immune markers were z-score standardized; odds ratios represent the change in odds of 30-day major adverse postoperative events (MAEs) per 1-SD increase in marker proportion. Each perioperative variable was added one at a time to the parsimonious base model adjusted for age, preoperative eGFR, and preoperative CRP.

## 4 Discussion

Our analysis of immune-cell abundances and phenotypes showed that CPB was accompanied by extensive perioperative immune alterations, which have been implicated in postoperative organ injury after cardiac surgery (20). In our cohort, higher counts of CD4⁺CD38⁺ T cells and CD274⁺ unswitched memory B cells at the end of CPB were associated with MAEs within 30 days after surgery. MAEs were also associated with abnormal activation and maturation of NK cells and their subpopulations at different time points and with a lower abundance of CD163⁺ classical monocytes but a higher abundance of CD284⁺ non-classical monocytes during rewarming, a pattern compatible with a more pro-inflammatory monocyte profile. Because preoperative characteristics differed between patients with and without MAEs, we performed parsimonious risk-adjusted analyses using Firth penalized logistic regression given the low event rate. After adjustment for age, preoperative eGFR, and preoperative CRP, the associations for CD284⁺ non-classical monocytes at rewarming and CD274⁺ unswitched memory B cells at the end of CPB remained statistically significant and were largely robust to additional adjustment for preoperative AST in sensitivity analyses. By contrast, the association for CD4⁺CD38⁺ T cells at the end of CPB was attenuated after adjusting for AST, suggesting that this signal may be more sensitive to covariate adjustment and limited statistical power. These findings are consistent with an association between perioperative immune disturbances and postoperative organ injury in this cohort and may help to identify candidate immune signatures associated with MAEs. They should nevertheless be interpreted cautiously given the limited sample size and number of events.

In a previous study from our group, we characterized perioperative immune-cell profiles in adults undergoing cardiac surgery with CPB and investigated their association with postoperative delirium (12). Although the two studies share a similar perioperative sampling framework, they address distinct clinical questions and endpoints. Both studies were derived from the same prospective database, with partial overlap in participants. The current study extends this prior work in two important ways. First, we focused on a broader and clinically more comprehensive endpoint: 30-day MAEs, capturing early postoperative multiorgan dysfunction beyond neurocognitive outcomes. Second, we emphasized the dynamic trajectories of immune-cell phenotypes across four perioperative time points and identified immune signatures involving T-cell activation/exhaustion, checkpoint-expressing B-cell subsets, natural killer-cell maturation, and monocyte polarization that were associated with MAEs.

We acknowledge that the composite endpoint included clinically diverse events across multiple organ systems. We selected this endpoint because postoperative morbidity after CPB is inherently multisystemic, and these complications are broadly linked by shared upstream processes, including systemic inflammation, ischemia–reperfusion injury, endothelial dysfunction, and perioperative immune dysregulation. At the same time, the diversity of these components should be considered when interpreting the findings. Accordingly, our results are best understood as reflecting associations with the overall burden of early postoperative multi-organ dysfunction rather than with any single complication. Given the limited number of events, analyses of individual endpoint components were underpowered and were therefore not a focus of the present study.

Our analyses showed that CPB was accompanied by a pronounced systemic inflammatory response, characterized by neutrophil-driven leukocytosis and lymphopenia. Neutrophil counts increased rapidly at the end of CPB and remained elevated for at least 24 h after surgery, which is consistent with previous studies demonstrating complement activation and pro-inflammatory cytokine release during CPB, followed by a peak in neutrophil count within 12–48 h after surgery (21, 22). In contrast, lymphocyte counts decreased during rewarming, recovered transiently at the end of CPB, and then declined substantially within 24 h after surgery. This pattern aligns with the well-established sequence of “inflammatory activation–immune suppression” observed after CPB (23). Both the NLR and SII peaked at 24 h after surgery (9, 19), further supporting the presence of an early postoperative immune imbalance characterized by concurrent pro-inflammatory activation and lymphocyte suppression. The ratio of CD4^+^ to CD8^+^ T cells significantly decreased at the end of CPB and increased 24 h after surgery, consistent with prior work (24). Taken together, these findings suggest that CPB is associated with concurrent inflammatory activation and immune suppression, manifesting as elevated neutrophils, NLR, and SII, whereas impaired cellular immunity manifests as lymphopenia and a reduced CD4⁺/CD8⁺ T-cell ratio. These observations are consistent with the idea that the perturbation of T cell-mediated immunity during CPB may be related to postoperative outcomes (25).

In our cohort, MAEs were associated with a markedly lower abundance of peripheral CD45RO⁺ T cells during rewarming. One possible interpretation is that this finding may reflect redistribution or altered effector/memory T-cell dynamics, although this cannot be confirmed from the present data. CD45RO⁺ effector/memory T cells perform immunosurveillance and facilitate tissue repair and resolution of inflammation (26). Consistently, abundance of peripheral CD45RO⁺ T cells declined during 6–24 h after surgery (27) and concomitantly increases in pericardial fluid (28). This apparent redistribution may be consistent with heightened local inflammation, but the present study cannot determine whether peripheral changes in CD45RO⁺ T cells reflect tissue recruitment, altered survival, or other perioperative processes. These observations are consistent with the association we observed between CD45RO⁺ T cell alterations and MAEs but do not establish causality.

MAEs were associated with a significantly higher abundance of CD38⁺ T cells at the end of CPB. Prior work suggests that CD38 is involved in reactive oxygen species generation (29), that CPB can upregulate CD38 expression (30), and that NAD⁺ depletion has been linked to oxidative stress and organ injury in other settings (31–33). These observations may be relevant to the association observed in our cohort. However, we did not assess CD38 enzymatic activity, NAD metabolism, oxidative stress, or downstream functional effects.

MAEs were associated with a significantly higher proportion of CD4⁺CD8⁺CD279⁺ T cells during rewarming. High expression of CD279, also known as PD-1, has been described as a marker of chronic activation or immune exhaustion (34), and its upregulation may represent a negative feedback mechanism to limit excessive inflammation (35). CPB is known to upregulate expression of CD279 and its ligand, CD274 or PD-L1 (34, 35). However, excessive inhibition through this pathway may be related to impaired tissue repair and adverse outcomes (36). In our cohort, patients with MAEs were associated with higher proportions of CD274⁺ unswitched memory B cells at the end of CPB. CD279 (PD-1) and its ligand CD274 (PD-L1) form a critical immune checkpoint pathway. CD274 expression on B cells has been linked to inhibitory immune signaling in previous studies (34). In our cohort, increased CD274⁺ unswitched memory B cells and CD279⁺ T-cell phenotypes were observed among patients with MAEs, which is consistent with possible involvement of the PD-1/PD-L1 pathway in perioperative immune regulation (37). However, our data do not directly assess pathway activation, cellular interactions, or functional consequences. Therefore, these findings should be interpreted as associations between checkpoint-related immune phenotypes and postoperative MAEs, without establishing causality.

In our cohort, MAEs were associated with lower peripheral abundance of NK cells both preoperatively and during CPB rewarming, which may be consistent with altered innate immune status (38). Indeed, MAEs in our study were associated with persistent downregulation of CD56^bri^CD335⁺ NK cells from preoperative through 24 h after surgery, just as a previous study reported CD335 downregulation in CPB-induced inflammation, which has been interpreted in previous studies as a marker of reduced NK-cell activity (39). On the other hand, MAEs in our cohort were associated with higher abundance of CD56^bri^ CD57⁺ NK cells across all time points. This subpopulation has a mature, inflammation-experienced phenotype that responds to interleukins-12 and -18 by producing interferon-*γ* (40), and its expansion has been reported in association with cardiovascular injury (40, 41). In addition, MAEs were also associated with higher proportions of CD314⁺ and CD56^dim^ CD314⁺ NK cells during rewarming. CD314, also known as NKG2D, has been associated with cytotoxic and pro-inflammatory signaling pathways in previous studies (42), including studies of organ injury affecting the kidneys, lungs and heart (42–44). However, the present study did not assess NK-cell cytotoxicity or downstream signaling. Taken together, these findings suggest that MAEs were associated with complex phenotypic alterations in the NK-cell compartment, characterized by concurrent changes in cell abundance, maturation-related phenotypes, and activation receptor expression, rather than by a simple unidirectional change in NK-cell phenotypes.

In our cohort, MAEs were associated with a lower proportion of CD163⁺ classical monocytes during rewarming, a subset generally considered to be involved in anti-inflammatory responses and tissue repair (45–47). This reduction may be consistent with altered anti-inflammatory or reparative monocyte phenotypes during the period of peak perioperative inflammatory stress. At the same time, patients with MAEs exhibited higher proportions of CD284⁺ (TLR4⁺) non-classical monocytes, a population that has been implicated in vascular surveillance and immune activation under physiological conditions (46). Under inflammatory conditions, TLR4 activation by mediators such as HMGB1 has been reported to be associated with oxidative stress and mitochondrial dysfunction (48, 49). However, our study did not measure HMGB1, oxidative stress, mitochondrial dysfunction, or TLR4 signaling activity. Taken together, these findings suggest that patients with MAEs exhibited a pattern of monocyte phenotypic differences characterized by reduced CD163⁺ classical monocytes and increased CD284⁺ non-classical monocytes. This pattern is compatible with, but does not prove, a relatively more pro-inflammatory monocyte phenotype during the rewarming phase of CPB.

Several limitations should be considered when interpreting these findings. First, this was a single-center study with a relatively small sample size and a limited number of patients who experienced MAEs, which may have reduced statistical power and limited generalizability. In particular, the ROC analyses were based on 10 MAEs. Therefore, the AUC estimates and any derived cutoffs may be unstable and should not be used for clinical risk prediction without external validation. Larger, preferably multicenter, cohorts are needed to validate these observations and strengthen external validity. Second, because this was an observational study based on peripheral immune phenotyping, causal relationships and underlying mechanisms cannot be inferred. Immune profiling was performed at relatively few time points and was not validated by functional assays or multi-omic approaches. Further studies are needed to determine whether the observed immune phenotypes contribute to, result from, or merely accompany postoperative MAEs. Third, to minimize the potential confounding effect of blood transfusions on immune-cell proportions, patients who received perioperative transfusions were excluded from the final analysis. Although this decision was methodologically justified, it may have introduced selection bias, as transfusion recipients tend to represent a clinically more complex population with greater intraoperative blood loss or hemodynamic instability. The immune profiles of transfused patients therefore remain unknown, and our findings may not be fully generalizable to higher-risk surgical populations. Fourth, all patients underwent CPB under a uniform protocol of moderate hypothermia (32–34 °C). Prior work suggests that, within this range, hypothermia versus near-normothermia may not markedly change systemic inflammatory marker profiles (50). However, this does not exclude more subtle effects of cooling and rewarming on specific immune-cell responses. Experimental data indicate that cooling and rewarming can still modulate monocyte activation alongside shear stress and blood–artificial surface interaction (51). Whether similar immune alterations would be observed under strictly normothermic CPB remains to be determined. Finally, although CPB duration and aortic cross-clamp time were examined as parsimonious indicators of procedural complexity, residual confounding related to procedural differences cannot be fully excluded because surgical procedure categories were not mutually exclusive and some were sparsely represented in this cohort. Because the number of MAEs was limited, we did not conduct comprehensive multivariable modeling across all measured immune subsets; instead, we focused on a small set of pre-specified markers to reduce overfitting and limit false-positive findings.

Despite these limitations, our study suggests that CPB may be associated with dynamic perioperative alterations in immune cell populations and that specific immune phenotypes were associated with MAEs in this cohort.

## 5 Conclusion

In this exploratory single-center cohort, cardiac surgery with CPB was associated with dynamic perioperative alterations in circulating immune cell populations. Patients who developed MAEs showed a more dysregulated perioperative immune profile, and several immune phenotypes were statistically associated with postoperative MAEs. These preliminary, hypothesis-generating findings require validation in larger multicenter studies and further investigation using functional or mechanistic approaches.

## Data Availability

The datasets presented in this article are not readily available because of ethical and privacy restrictions. Requests to access the datasets should be directed to Jing Lin, linjing@wchscu.cn.

## References

[1] Oczenski W Krenn H Jilch R Watzka H Waldenberger F Koller U HLA-DR as a marker for increased risk for systemic inflammation and septic complications after cardiac surgery. Intensive Care Med. (2003) 29:1253–7. 10.1007/s00134-003-1826-8 12802492

[2] Chenouard A Braudeau C Cottron N Bourgoin P Salabert N Roquilly A HLA-DR expression in neonates after cardiac surgery under cardiopulmonary bypass: a pilot study. Intensive Care Med Exp. (2018) 6:1. 10.1186/s40635-017-0166-x 29327145 PMC5764905

[3] Squiccimarro E Stasi A Lorusso R Paparella D. Narrative review of the systemic inflammatory reaction to cardiac surgery and cardiopulmonary bypass. Artif Organs. (2022) 46:568–77. 10.1111/aor.14171 35061922 PMC9303696

[4] Banerjee D Feng J Sellke FW. Strategies to attenuate maladaptive inflammatory response associated with cardiopulmonary bypass. Front Surg. (2024) 11:1224068. 10.3389/fsurg.2024.1224068 39022594 PMC11251955

[5] Rinder CS Fontes M Mathew JP Rinder HM Smith BR, Multicenter Study of Perioperative Ischemia Research Group. Neutrophil CD11b upregulation during cardiopulmonary bypass is associated with postoperative renal injury. Ann Thorac Surg. (2003) 75:899–905. 10.1016/S0003-4975(02)04490-9 12645714

[6] Mayer D Altvater M Schenz J Arif R Karck M Leuschner F Monocyte metabolism and function in patients undergoing cardiac surgery. Front Cardiovasc Med. (2022) 9:853967. 10.3389/fcvm.2022.853967 35935635 PMC9347004

[7] Broeders W Van Tuijl J Duindam HB Peters Van Ton AM Noz MP Pickkers P Long-term monocyte activation after coronary artery bypass grafting: an exploratory prospective observational study. Immunol Lett. (2024) 270:106941. 10.1016/j.imlet.2024.106941 39489184

[8] Flores-Gomez D Bekkering S Netea MG Riksen NP. Trained immunity in atherosclerotic cardiovascular disease. Arterioscler Thromb Vasc Biol. (2021) 41:62–9. 10.1161/ATVBAHA.120.314216 33147995

[9] Gawdat K Legere S Wong C Myers T Marshall JS Hassan A Changes in circulating monocyte subsets (CD16 expression) and neutrophil-to-lymphocyte ratio observed in patients undergoing cardiac surgery. Front Cardiovasc Med. (2017) 4:12. 10.3389/fcvm.2017.00012 28361055 PMC5350132

[10] Weedle RC Da Costa M Veerasingam D Soo AWS. The use of neutrophil lymphocyte ratio to predict complications post cardiac surgery. Ann Transl Med. (2019) 7:778. 10.21037/atm.2019.11.17 32042794 PMC6989975

[11] Wang T Yao Y Sun J Wu J Liao X Yang P Single-cell immune landscape of acute kidney injury after cardiac surgery with cardiopulmonary bypass. Ren Fail. (2025) 47:2581506. 10.1080/0886022X.2025.2581506 41199602 PMC12599361

[12] Wu J Cheng Z Liao X Yang P Wu Q Wang T Perioperative profiles of immune cells in patients with postoperative delirium after cardiac surgery with cardiopulmonary bypass. Biomedicines. (2025) 13:2962. 10.3390/biomedicines13122962 41462974 PMC12730709

[13] von Elm E Altman DG Egger M Pocock SJ Gøtzsche PC Vandenbroucke JP Strengthening the reporting of observational studies in epidemiology (STROBE) statement: guidelines for reporting observational studies. Br Med J. (2007) 335:806–8. 10.1136/bmj.39335.541782.AD 17947786 PMC2034723

[14] Wang T Yao Y Sun J Wu J Liao X Meng W Neutrophil activation is correlated with acute kidney injury after cardiac surgery under cardiopulmonary bypass. Chin J Blood Transfus. (2025) 38:358–67. 10.13303/j.cjbt.issn.1004-549x.2025.03.009

[15] Zheng Y Ran Y Wu J Yang P Liao X Zhang J *In vitro* validation of a novel disposable remover to remove activated leukocytes generated during cardiopulmonary bypass: a pilot study. J Inflammation Res. (2025) 18:5355–70. 10.2147/JIR.S503575 PMC12015734 40270561

[16] Stoppe C McDonald B Meybohm P Christopher KB Fremes S Whitlock R Effect of high-dose selenium on postoperative organ dysfunction and mortality in cardiac surgery patients: the SUSTAIN CSX randomized clinical trial. JAMA Surg. (2023) 158:235–44. 10.1001/jamasurg.2022.6855 36630120 PMC9857635

[17] Landis RC Brown JR Fitzgerald D Likosky DS Shore-Lesserson L Baker RA Attenuating the systemic inflammatory response to adult cardiopulmonary bypass: a critical review of the evidence base. J Extra Corpor Technol. (2014) 46:197–211. 10.1051/ject/201446197 26357785 PMC4566828

[18] Jammer I Wickboldt N Sander M Smith A Schultz MJ Pelosi P Standards for definitions and use of outcome measures for clinical effectiveness research in perioperative medicine: European perioperative clinical outcome (EPCO) definitions: a statement from the ESA-ESICM joint taskforce on perioperative outcome measures. Eur J Anaesthesiol. (2015) 32:88–105. 10.1097/EJA.0000000000000118 25058504

[19] Staicu R-E Cozlac A-R Sintean ME Negru AG Gorun F Ciurescu S Inflammatory biomarkers for predicting postoperative atrial fibrillation in cardiac surgery. J Med Life. (2025) 18:494–508. 10.25122/jml-2025-0085 40599146 PMC12207702

[20] Day JRS Taylor KM. The systemic inflammatory response syndrome and cardiopulmonary bypass. Int J Surg. (2005) 3:129–40. 10.1016/j.ijsu.2005.04.002 17462274

[21] Lesouhaitier M Gregoire M Gacouin A Coirier V Frerou A Piau C Neutrophil function and bactericidal activity against Staphylococcus aureus after cardiac surgery with cardiopulmonary bypass. J Leukoc Biol. (2022) 111:867–76. 10.1002/JLB.5AB1219-737RR 34425029

[22] Mossanen JC Jansen TU Pracht J Liepelt A Buendgens L Stoppe C Elevated circulating CD14^++^CD16^+^ intermediate monocytes are independently associated with extracardiac complications after cardiac surgery. Sci Rep. (2020) 10:947. 10.1038/s41598-020-57700-9 31969629 PMC6976615

[23] Rinder CS Mathew JP Rinder HM Tracey JB Davis E Smith BR. Lymphocyte and monocyte subset changes during cardiopulmonary bypass: effects of aging and gender. J Lab Clin Med. (1997) 129:592–602. 10.1016/S0022-2143(97)90193-1 9178725

[24] Sindera P Zawidlak-Wegrzynska B Kucewicz-Czech E. Potential use of immunological indices as predictors of bacterial infections following cardiopulmonary bypass surgery in elderly patients. J Biomed Res Environ Sci. (2025) 6:297–308. 10.37871/jbres2084

[25] Luo W Sun J-J Tang H Fu D Hu Z-L Zhou H-Y Association of apoptosis-mediated CD4+ T lymphopenia with poor outcome after type A aortic dissection surgery. Front Cardiovasc Med. (2021) 8:747467. 10.3389/fcvm.2021.747467 34869652 PMC8632808

[26] Sallusto F Geginat J Lanzavecchia A. Central memory and effector memory T cell subsets: function, generation, and maintenance. Annu Rev Immunol. (2004) 22:745–63. 10.1146/annurev.immunol.22.012703.104702 15032595

[27] Iske J Wiegmann B Ius F Chichelnitskiy E Ludwig K Kühne JF Immediate major dynamic changes in the T- and NK-cell subset composition after cardiac transplantation. Eur J Immunol. (2023) 53:e2250097. 10.1002/eji.202250097 37119053

[28] Gilicze O Simon D Farkas N Lantos M Jancso G Berki T Characterization of lymphocyte subpopulations and cardiovascular markers in pericardial fluid of cardiac surgery patients. Clin Hemorheol Microcirc. (2020) 73:579–90. 10.3233/CH-190594 31156149

[29] Gouchoe DA Vijayakumar A Aly AH Cui EY Essandoh M Gumina RJ The role of CD38 in ischemia reperfusion injury in cardiopulmonary bypass and thoracic transplantation: a narrative review. J Thorac Dis. (2023) 15:5736–49. 10.21037/jtd-23-725 37969313 PMC10636473

[30] Reyes LA Boslett J Varadharaj S De Pascali F Hemann C Druhan LJ Depletion of NADP(H) due to CD38 activation triggers endothelial dysfunction in the postischemic heart. Proc Natl Acad Sci USA. (2015) 112:11648–53. 10.1073/pnas.1505556112 26297248 PMC4577172

[31] Guan X-H Liu X-H Hong X Zhao N Xiao Y-F Wang L-F CD38 deficiency protects the heart from ischemia/reperfusion injury through activating SIRT1/FOXOs-mediated antioxidative stress pathway. Oxid Med Cell Longev. (2016) 2016:7410257. 10.1155/2016/7410257 27547294 PMC4983367

[32] Wu M-Y Yiang G-T Liao W-T Tsai AP-Y Cheng Y-L Cheng P-W Current mechanistic concepts in ischemia and reperfusion injury. Cell Physiol Biochem. (2018) 46:1650–67. 10.1159/000489241 29694958

[33] Wang L-F Cao Q Wen K Xiao Y-F Chen T-T Guan X-H CD38 deficiency alleviates D-galactose-induced myocardial cell senescence through NAD+/Sirt1 signaling pathway. Front Physiol. (2019) 10:1125. 10.3389/fphys.2019.01125 31551807 PMC6735286

[34] Sharpe AH Pauken KE. The diverse functions of the PD1 inhibitory pathway. Nat Rev Immunol. (2018) 18:153–67. 10.1038/nri.2017.108 28990585

[35] Lesouhaitier M Belicard F Tadié J-M. Cardiopulmonary bypass and VA-ECMO induced immune dysfunction: common features and differences, a narrative review. Crit Care. (2024) 28:300. 10.1186/s13054-024-05058-z 39256830 PMC11389086

[36] Miyazaki S Fujisue K Yamanaga K Sueta D Usuku H Tabata N Prognostic significance of soluble PD-L1 on cardiovascular outcomes in patients with coronary artery disease. J Atheroscler Thromb. (2024) 31:355–67. 10.5551/jat.64183 37793811 PMC10999719

[37] Garcia-Lacarte M Grijalba SC Melchor J Arnaiz-Leché A Roa S. The PD-1/PD-L1 checkpoint in normal germinal centers and diffuse large B-cell lymphomas. Cancers (Basel). (2021) 13:4683. 10.3390/cancers13184683 34572910 PMC8471895

[38] Sun JC Lanier LL. NK cell development, homeostasis and function: parallels with CD8+ T cells. Nat Rev Immunol. (2011) 11:645–57. 10.1038/nri3044 21869816 PMC4408539

[39] Wu J Cheng Z Yang S Wu Q Yang P Liao X Specific immune landscape of heatstroke distinguished from sepsis and aseptic inflammation. Int J Med Sci. (2025) 22:1450–64. 10.7150/ijms.108212 40084252 PMC11898847

[40] Shin E Bak SH Park T Kim JW Yoon S-R Jung H Understanding NK cell biology for harnessing NK cell therapies: targeting cancer and beyond. Front Immunol. (2023) 14:1192907. 10.3389/fimmu.2023.1192907 37539051 PMC10395517

[41] Tsang HW Kwan MYW Chua GT Tsao SSL Wong JSC Tung KTS The central role of natural killer cells in mediating acute myocarditis after mRNA COVID-19 vaccination. Med. (2024) 5:335–347.e3. 10.1016/j.medj.2024.02.008 38521068

[42] Matsumoto K Obana M Kobayashi A Kihara M Shioi G Miyagawa S Blockade of NKG2D/NKG2D ligand interaction attenuated cardiac remodelling after myocardial infarction. Cardiovasc Res. (2019) 115:765–75. 10.1093/cvr/cvy254 30307485

[43] Calabrese DR Aminian E Mallavia B Liu F Cleary SJ Aguilar OA Natural killer cells activated through NKG2D mediate lung ischemia-reperfusion injury. J Clin Invest. (2021) 131:e137047. 10.1172/JCI137047 33290276 PMC7852842

[44] Cantoni C Granata S Bruschi M Spaggiari GM Candiano G Zaza G. Recent advances in the role of natural killer cells in acute kidney injury. Front Immunol. (2020) 11:1484. 10.3389/fimmu.2020.01484 32903887 PMC7438947

[45] Kratofil RM Kubes P Deniset JF. Monocyte conversion during inflammation and injury. Arterioscler Thromb Vasc Biol. (2017) 37:35–42. 10.1161/ATVBAHA.116.308198 27765768

[46] Thomas G Tacke R Hedrick CC Hanna RN. Nonclassical patrolling monocyte function in the vasculature. Arterioscler Thromb Vasc Biol. (2015) 35:1306–16. 10.1161/ATVBAHA.114.304650 25838429 PMC4441550

[47] Philippidis P Mason JC Evans BJ Nadra I Taylor KM Haskard DO Hemoglobin scavenger receptor CD163 mediates interleukin-10 release and heme oxygenase-1 synthesis: antiinflammatory monocyte-macrophage responses *in vitro*, in resolving skin blisters *in vivo*, and after cardiopulmonary bypass surgery. Circ Res. (2004) 94:119–26. 10.1161/01.RES.0000109414.78907.F9 14656926

[48] Li Z Fan EK Liu J Scott MJ Li Y Li S Cold-inducible RNA-binding protein through TLR4 signaling induces mitochondrial DNA fragmentation and regulates macrophage cell death after trauma. Cell Death Dis. (2017) 8:e2775. 10.1038/cddis.2017.187 28492546 PMC5584526

[49] Deng Y Hou L Xu Q Liu Q Pan S Gao Y Cardiopulmonary bypass induces acute lung injury via the high-mobility group box 1/toll-like receptor 4 pathway. Dis Markers. (2020) 2020:8854700. 10.1155/2020/8854700 33062073 PMC7532999

[50] Schmitt KRL Fedarava K Justus G Redlin M Böttcher W Delmo Walter EM Hypothermia during cardiopulmonary bypass increases need for inotropic support but does not impact inflammation in children undergoing surgical ventricular septal defect closure. Artif Organs. (2016) 40:471–9. 10.1111/aor.12587 26581834

[51] Tu LN Hsieh L Kajimoto M Charette K Kibiryeva N Forero A Shear stress associated with cardiopulmonary bypass induces expression of inflammatory cytokines and necroptosis in monocytes. JCI insight. (2021) 6:e141341. 10.1172/jci.insight.141341 33232305 PMC7821587

