# Supplementary material for: Perioperative immune dynamics during cardiopulmonary bypass and association with major adverse postoperative events
Source: Front Cardiovasc Med. 2026 May 26;13:1811178. doi: 10.3389/fcvm.2026.1811178 (PMC13246431; doi:10.3389/fcvm.2026.1811178)
Supplement: Supplementary file 1 [file Datasheet1.docx]

***Supplementary Material***

Supplement to: Zhiyuan Cheng, Xinyi Liao, Juan Wu, et al. **Perioperative immune dynamics during cardiopulmonary bypass and association with major adverse postoperative events**

This material has been provided by the authors to give readers additional information about the work.

**Supplementary Material**

Supplement to: **Perioperative immune dynamics during cardiopulmonary bypass and association with major adverse postoperative events**

Zhiyuan Cheng, Xinyi Liao, Juan Wu, Ping Yang, Qinjuan Wu, Zongcheng Tang, Yishun Wang, Wentong Meng, Lei Du, Jing Lin

Contents

Supplementary Figure S1……………………………………………………………………….………...3

Supplementary Table S1……………………………………………………………………….………....4

Supplementary Definitions………………………………...………………………………….………….6

Supplementary References………………………………...………………………………….………….7

Supplementary Figure S2………………………………………………………………………………...9

Supplementary Figure S3………………………………………………………………………………...10

Supplementary Figure S4………………………………………………………………………………...11

Supplementary Figure S5………………………………………………………………………………...12

Supplementary Table S2……………………………………………………………………….………....12


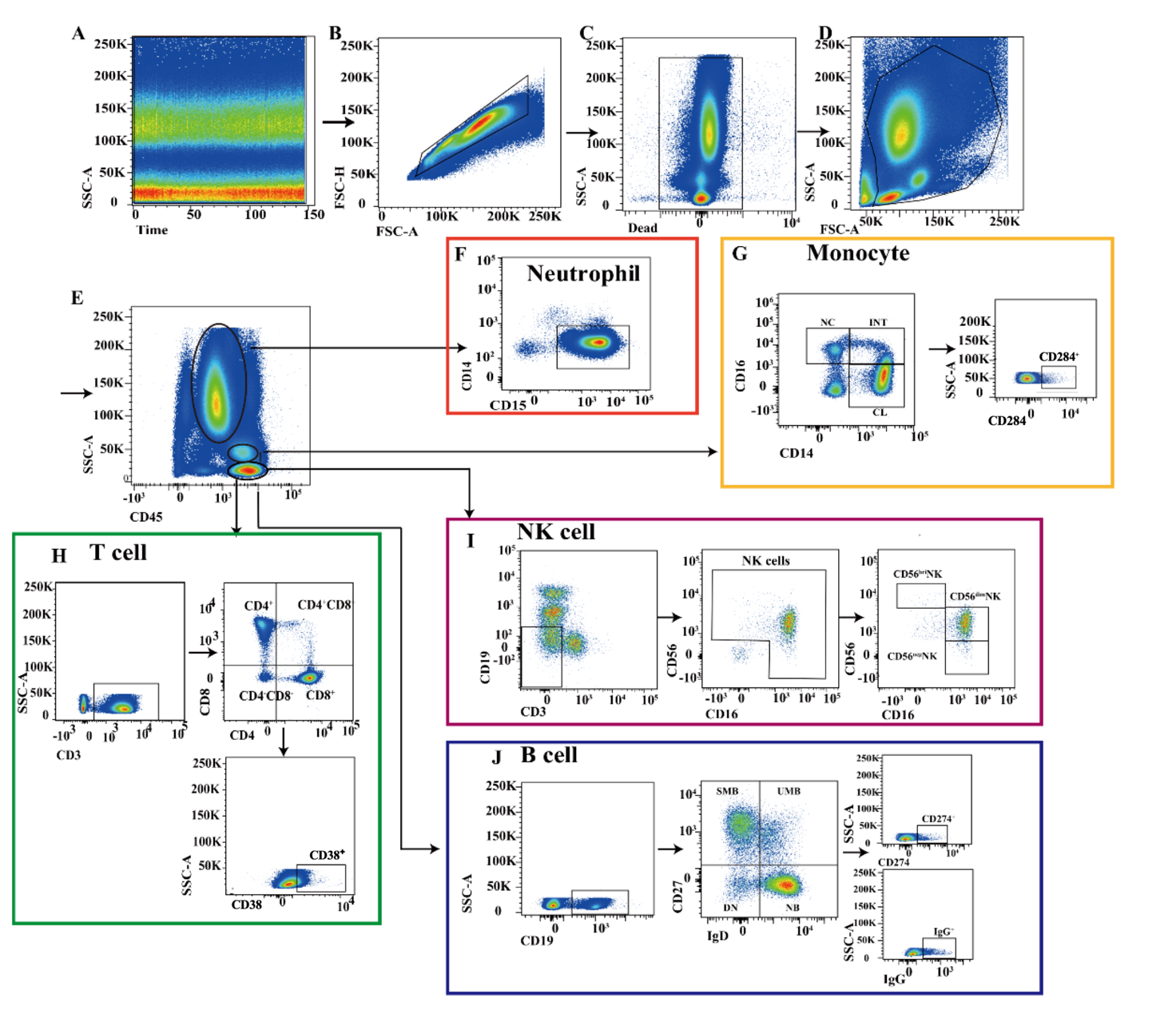


Supplementary Figure S1 Representative gating strategy for identification of circulating immune cell populations and subpopulations. (A) Assessment of acquisition stability using Time versus SSC-A parameters. (B) Doublet exclusion using FSC-A versus FSC-H gating. (C) Dead-cell exclusion using viability dye (Dead) and SSC-A. (D) Initial gating based on FSC-A and SSC-A to distinguish major leukocyte populations. (E) Identification of CD45⁺ leukocytes and preliminary separation of granulocytes, monocytes, and lymphocytes based on scatter properties. (F) Identification of neutrophils based on CD15 and CD14 expression. (G) Identification of monocyte subsets based on CD14 and CD16 expression, including classical (CL), intermediate (INT), and non-classical (NC) monocytes, followed by phenotypic marker analysis (e.g., CD80). (H) Identification of T cells (CD3⁺) and further subdivision into CD4⁺ and CD8⁺ subsets, followed by assessment of activation markers (e.g., CD69). (I) Identification of natural killer (NK) cells as CD3⁻CD19⁻ lymphocytes and further subdivision based on CD56 and CD16 expression into CD56dim CD56brisubsets. (J) Identification of B cells (CD19⁺) and further classification into switched memory (SMB), unswitched memory (UMB), naïve B cells (NB), and double-negative (DN) subsets based on IgD and CD27 expression, followed by phenotypic marker analysis (e.g., CD274 and IgG). All samples were analyzed using a standardized gating template, and representative plots from one individual are shown.

Supplementary Table S1 Antibody panels used for multicolour flow cytometry.

| Panel | Marker | Fluorochrome | Clone | Manufacturer | Volume per test | Purpose |
| --- | --- | --- | --- | --- | --- | --- |
| T-cell | CD45 | BV605 | HI30 | BD Pharmingen | 2 μL | Identification |
| T-cell | CD3 | PerCP-Cy5.5 | UCHT1 | BD Pharmingen | 3 μL | Identification |
| T-cell | CD4 | APC-Cy7 | RPA-T4 | BD Pharmingen | 2 μL | Identification |
| T-cell | CD8 | BV510 | SK1 | BD Pharmingen | 2 μL | Subsetting |
| T-cell | CD45RO | APC | UCHL1 | BD Pharmingen | 2 μL | Subsetting |
| T-cell | CD69 | PE | FN50 | BD Pharmingen | 2 μL | Activation/phenotyping |
| T-cell | CD38 | FITC | HIT2 | BD Pharmingen | 2 μL | Activation/phenotyping |
| T-cell | CD28 | PE-Cy7 | CD28.2 | BD Pharmingen | 2 μL | Activation/phenotyping |
| T-cell | CD279 (PD-1) | BV421 | NA | BD Pharmingen | 2 μL | Activation/phenotyping |
| T-cell | FVS700 | — | — | BD Pharmingen | 1 μL | Viability control |
| B-cell | CD45 | BV605 | NA | BD Pharmingen | 2 μL | Identification |
| B-cell | CD19 | BV510 | NA | BD Pharmingen | 2 μL | Identification |
| B-cell | CD27 | BV421 | NA | BD Pharmingen | 2 μL | Subsetting |
| B-cell | IgD | APC-H7 | NA | BD Pharmingen | 2 μL | Subsetting |
| B-cell | IgM | PerCP-Cy5.5 | NA | BD Pharmingen | 2 μL | Activation/phenotyping |
| B-cell | IgG | PE | NA | BD Pharmingen | 4 μL | Activation/phenotyping |
| B-cell | CD38 | FITC | NA | BD Pharmingen | 2 μL | Activation/phenotyping |
| B-cell | CD274 (PD-L1) | PE-Cy7 | NA | BD Pharmingen | 2 μL | Activation/phenotyping |
| B-cell | CD80 | APC | NA | BioLegend | 3 μL | Activation/phenotyping |
| B-cell | FVS700 | — | — | BD Pharmingen | 1 μL | Viability control |
| Monocyte | CD45 | BV605 | NA | BD Pharmingen | 2 μL | Identification |
| Monocyte | CD14 | FITC | NA | BD Pharmingen | 4 μL | Identification |
| Monocyte | CD16 | APC-H7 | NA | BD Pharmingen | 2 μL | Identification/subsetting |
| Monocyte | HLA-DR | PE | NA | BD Pharmingen | 4 μL | Activation/phenotyping |
| Monocyte | CD80 | PerCP-Cy5.5 | NA | BD Pharmingen | 2 μL | Activation/phenotyping |
| Monocyte | CD40 | BV510 | NA | BD Pharmingen | 2 μL | Activation/phenotyping |
| Monocyte | CD163 | Alexa Fluor 647 | NA | BD Pharmingen | 2 μL | Activation/phenotyping |
| Monocyte | CD274 (PD-L1) | PE-Cy7 | NA | BD Pharmingen | 2 μL | Activation/phenotyping |
| Monocyte | CD284 (TLR4) | BV421 | NA | BD Pharmingen | 2 μL | Activation/phenotyping |
| Monocyte | FVS700 | — | — | BD Pharmingen | 1 μL | Viability control |
| NK-cell | CD45 | BV605 | NA | BD Pharmingen | 2 μL | Identification |
| NK-cell | CD3 | PerCP-Cy5.5 | NA | BD Pharmingen | 3 μL | Exclusion |
| NK-cell | CD16 | APC-H7 | NA | BD Pharmingen | 2 μL | Subsetting |
| NK-cell | CD56 | PE-Cy7 | NCAM-1 | BD Pharmingen | 2 μL | Subsetting |
| NK-cell | CD57 | BV421 | NK-1 | BD Pharmingen | 2 μL | Activation/phenotyping |
| NK-cell | CD335 | BV510 | NA | BD Pharmingen | 2 μL | Activation/phenotyping |
| NK-cell | CD314 | APC | NA | BD Pharmingen | 2 μL | Activation/phenotyping |
| NK-cell | CD127 | PE | NA | BD Pharmingen | 2 μL | Activation/phenotyping |
| NK-cell | FVS700 | — | — | BD Pharmingen | 1 μL | Viability control |
| Granulocyte | CD45 | BV605 | NA | BD Pharmingen | 2 μL | Identification |
| Granulocyte | CD15 | BV510 | NA | BD Pharmingen | 2 μL | Identification |
| Granulocyte | CD14 | FITC | NA | BD Pharmingen | 4 μL | Reference |
| Granulocyte | CD123 | BV421 | NA | BD Pharmingen | 2 μL | Phenotyping |
| Granulocyte | Siglec-8 | PE | NA | BioLegend | 1 μL | Phenotyping |
| Granulocyte | CD54 | PerCP-Cy5.5 | NA | BioLegend | 2 μL | Activation/phenotyping |
| Granulocyte | CD11b | PE-Cy7 | NA | BD Pharmingen | 2 μL | Activation/phenotyping |
| Granulocyte | CD181 | APC | NA | BD Pharmingen | 4 μL | Activation/phenotyping |
| Granulocyte | CD64 | APC-H7 | NA | BD Pharmingen | 2 μL | Activation/phenotyping |
| Granulocyte | FVS700 | — | — | BD Pharmingen | 1 μL | Viability control |

**Abbreviations:** FVS, Fixable Viability Stain; PD-1, programmed cell death protein 1; PD-L1, programmed death-ligand 1; TLR4, toll-like receptor 4. Clone information was recorded as “NA” when it was not retrievable from the original laboratory records. Antibodies were grouped according to their primary analytical purpose: “Identification” indicates lineage markers used to define major immune cell populations; “Subsetting” indicates markers used for classification of cell subsets; “Activation/phenotyping” indicates markers used to assess activation status, co-stimulatory/co-inhibitory signaling, or functional phenotypes. Fixable Viability Stain 700 was used to exclude dead cells. Each antibody panel was applied separately to independent aliquots of the same sample.

Supplementary Definitions

Major adverse postoperative events (MAEs) were defined as follows:

1. Acute kidney injury was defined as any of the following(1):
   1. increase in serum creatinine by ≥ 0.3 mg/dL (≥26.5 μmol/L) within 48 h,
   2. increase in serum creatinine to ≥ 1.5 times baseline during the previous week,
   3. urine volume < 0.5 ml/kg/h for 6 h.
2. Neurological dysfunction was defined as occurrence of delirium, agitation, trance, dullness, or transient focal neurological deficits without any evidence of new structural abnormalities on computed tomography or magnetic resonance imaging(2)(3).
3. Mild liver injury was defined as elevation in alanine transaminase level to more than 3 times the upper reference limit (URL) (50 IU/L). Severe liver injury was defined as elevation in alanine transaminase level to at least 8 times the URL (≥400 IU/L), or as the combination of elevation in alanine transaminase to more than 3 times the URL together with elevation of total bilirubin to more than twice the URL. Patients who had mild liver injury before surgery and severe injury afterward were diagnosed with newly developed severe liver injury(4).
4. Cardiovascular complications were defined to include low cardiac output syndrome, malignant arrhythmias, and myocardial infarction.
5. Low cardiac output syndrome was defined as decreased cardiac output and hypoperfusion of peripheral organs as manifested in either one of the following two conditions(5)(6):
6. Requirement for two or more inotropic agents [epinephrine, milrinone, dobutamine, or high-dose dopamine (> 5 μg • kg^−1^ • min^−1^)] at 24-48 h postoperatively in the presence of at least one of the following conditions:
7. documented low cardiac output (< 2.0 L • min^−1^ • m^−2^) after cardiopulmonary bypass and/or immediately after surgery in the intensive care unit.
8. left and/or right ventricular dysfunction based on intraoperative echocardiography after cardiopulmonary bypass; or
9. chart documentation of postoperative cardiac insufficiency or cardiogenic shock.

A requirement for vasopressor support (norepinephrine, vasopressin, phenylephrine) without the use of two or more inotropic agents as described in point (1-i) above was not considered low cardiac output syndrome.

1. Use of mechanical circulatory support with extracorporeal membrane oxygenation, a left ventricular assist device, or intra-aortic balloon pump within the first five days after surgery to treat refractory cardiac failure despite the use of multiple inotropic agents.
2. Malignant arrhythmias were defined as rhythmic disturbances causing hemodynamic instability within a short period, leading to syncope or even sudden cardiac death. These disturbances could include(7): ventricular tachycardia, ventricular fibrillation, and cardiac arrest, defined as abrupt loss of consciousness in the absence of a systemic circulatory pulse.
3. Myocardial Infarction: The diagnostic criteria of new-onset myocardial infarction after cardiac surgery include clinical evidence of myocardial injury and myocardial ischemia as follows(8)(9)(10)(11):
4. Early-onset myocardial infarction (occurring during or within 72 hours after cardiac surgery):
5. Evidence of myocardial injury: For patients with normal preoperative troponin levels, if within 72 hours postoperatively the serum troponin value exceeds more than 100 times the upper reference limit (URL), or for patients with the preoperative troponin levels already above the URL but stable (≤20%) or decreasing, if the postoperative serum troponin increases more than 20% of the baseline, and must exceed 10 folds the URL.
6. Evidence of myocardial ischemia (including at least one of the following): New-onset left bundle branch block; Development of new pathological Q waves on the ECG; Imaging evidence (cardiac magnetic resonance or echocardiography) of new loss of viable myocardium or new regional wall motion abnormality.
7. Identification of a coronary thrombus by angiography or autopsy.
8. Late-onset myocardial infarction (within 72 hours to 30 days post-cardiac surgery):
9. Detection of an increase and/or decrease in troponin levels, with at least one value above 1-fold the URL, and including at least one piece of clinical evidence of myocardial ischemia:

- Symptoms of myocardial ischemia, such as chest pain or hemodynamic fluctuations that cannot be explained by other proven clinical conditions (e.g. pulmonary embolism, myocarditis);
- New ischemic changes on electrocardiography, defined as new or presumed significant ST-segment or T-wave changes on the electrocardiogram, or new-onset left bundle branch block.
- Development of new pathological Q waves on electrocardiography.
- Imaging evidence of new loss of viable myocardium or new regional wall motion abnormality through cardiac magnetic resonance imaging or echocardiography.

1. Identification of a coronary thrombus by angiography or autopsy.
2. Respiratory dysfunction was defined as severe respiratory dysfunction that prevented normal breathing at rest, leading to hypoxemia or carbon dioxide retention and resulting in a range of physiological dysfunctions and metabolic disorders(12) .

Supplementary References

1. Khwaja A. KDIGO clinical practice guidelines for acute kidney injury. *Nephron Clin Pract* (2012) 120:c179-184. doi: 10.1159/000339789

2. Su X, Meng Z-T, Wu X-H, Cui F, Li H-L, Wang D-X, Zhu X, Zhu S-N, Maze M, Ma D. Dexmedetomidine for prevention of delirium in elderly patients after non-cardiac surgery: a randomised, double-blind, placebo-controlled trial. *Lancet* (2016) 388:1893–1902. doi: 10.1016/S0140-6736(16)30580-3

3. Comas GM, Leshnower BG, Halkos ME, Thourani VH, Puskas JD, Guyton RA, Kilgo PD, Chen EP. Acute type a dissection: impact of antegrade cerebral perfusion under moderate hypothermia. *Ann Thorac Surg* (2013) 96:2135–2141. doi: 10.1016/j.athoracsur.2013.06.085

4. Raman JS, Kochi K, Morimatsu H, Buxton B, Bellomo R. Severe ischemic early liver injury after cardiac surgery. *Ann Thorac Surg* (2002) 74:1601–1606. doi: 10.1016/S0003-4975(02)03877-8

5. Duncan AE, Kartashov A, Robinson SB, Randall D, Zhang K, Luber J, James RA, Halvorson S, Bokesch P. Risk factors, resource use, and cost of postoperative low cardiac output syndrome. *J Thorac Cardiovasc Surg* (2022) 163:1890-1898.e10. doi: 10.1016/j.jtcvs.2020.06.125

6. Hijazi RM, Sessler DI, Liang C, Rodriguez-Patarroyo FA, Soltesz EG, Duncan AE. Association between In-hospital Mortality and Low Cardiac Output Syndrome with Morning versus Afternoon Cardiac Surgery: A Retrospective Cohort Study. *Anesthesiology* (2021) 134:552. doi: 10.1097/ALN.0000000000003728

7. Epstein AE, DiMarco JP, Ellenbogen KA, Estes NAM, Freedman RA, Gettes LS, Gillinov AM, Gregoratos G, Hammill SC, Hayes DL, et al. ACC/AHA/HRS 2008 guidelines for device-based therapy of cardiac rhythm abnormalities. *J Am Coll Cardiol* (2008) 51:e1–e62. doi: 10.1016/j.jacc.2008.02.032

8. Devereaux PJ, Whitlock R, Lamy A. Perioperative Myocardial Injury/Infarction After Cardiac Surgery: The Diagnostic Criteria Need to Change∗. *J Am Coll Cardiol* (2023) 82:1313–1315. doi: 10.1016/j.jacc.2023.08.001

9. Thygesen K, Alpert JS, Jaffe AS, Chaitman BR, Bax JJ, Morrow DA, White HD. Fourth Universal Definition of Myocardial Infarction (2018). *J Am Coll Cardiol* (2018) 72:2231–2264. doi: 10.1016/j.jacc.2018.08.1038

10. Devereaux PJ, Lamy A, Chan MTV, Allard RV, Lomivorotov VV, Landoni G, Zheng H, Paparella D, McGillion MH, Belley-Côté EP, et al. High-sensitivity troponin I after cardiac surgery and 30-day mortality. *N Engl J Med* (2022) 386:827–836. doi: 10.1056/NEJMoa2000803

11. Nicolas J, Soriano K, Salter B, Gross CR, Oloomi M, Dangas G. Myocardial infarction after cardiac surgery: When to intervene? *J Thorac Cardiovasc Surg* (2023) 165:1195–1201. doi: 10.1016/j.jtcvs.2021.08.074

12. Acute Respiratory Distress Syndrome: The Berlin Definition. *JAMA* (2012) 307:2526–2533. doi: 10.1001/jama.2012.5669


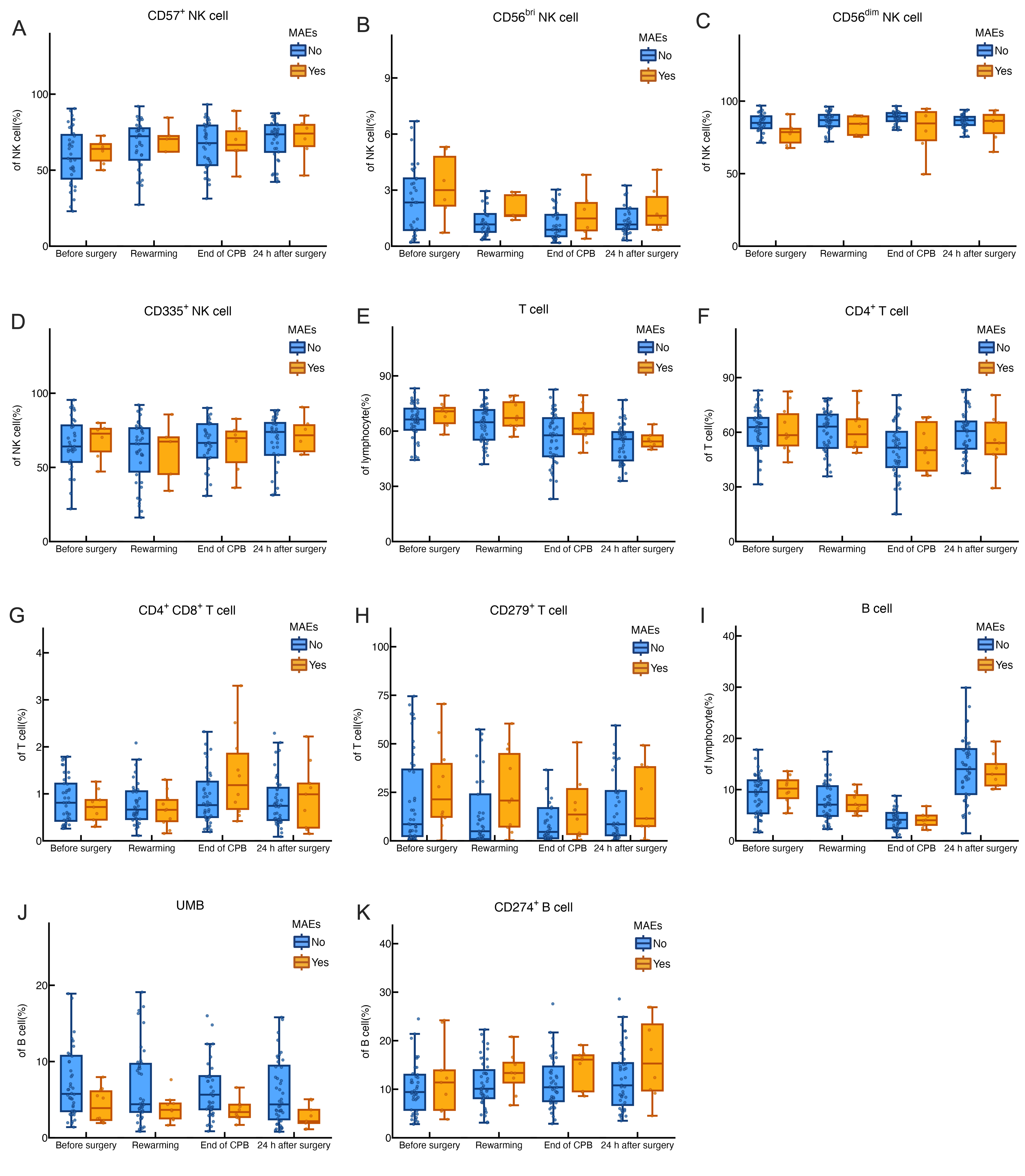


Supplementary Figure S2 (A-K) Comparison of the proportions of natural killer (NK) cell subpopulations, total T cells, T-cell subpopulations, total B cells, and B-cell subpopulations between the 10 patients who experienced major adverse postoperative events (MAEs) within 30 days after surgery and the 50 patients who did not. UMB, unswitched memory B cells.


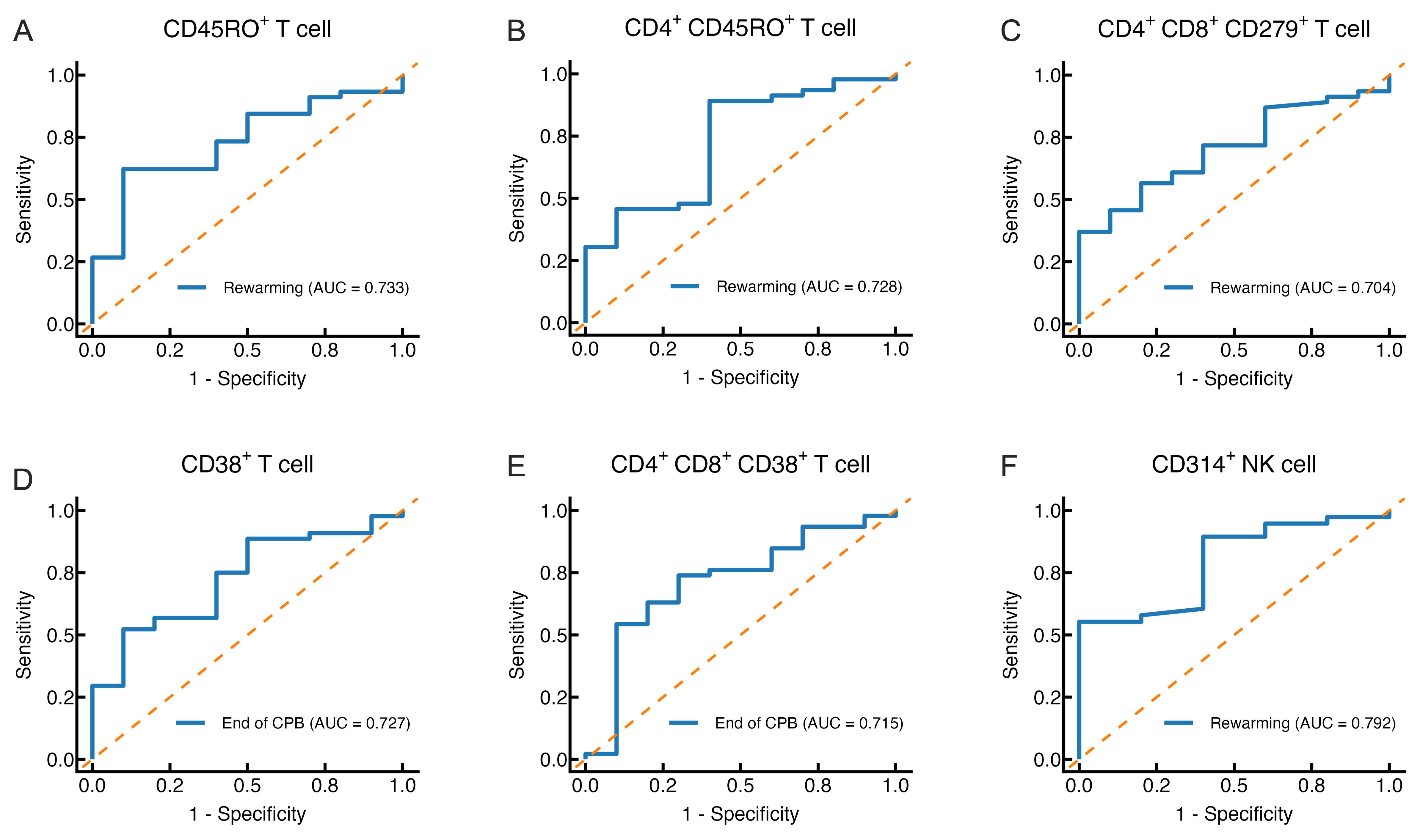


Supplementary Figure S3 (A-F) Exploratory receiver operating characteristic curves (ROC) describing within-cohort discrimination between patients with and without major adverse postoperative events (MAEs) based on the proportions of T-cell subpopulations and CD314⁺ natural killer (NK) cells at rewarming or the end of cardiopulmonary bypass (CPB). The area under the curve (AUC) is shown at the lower right.


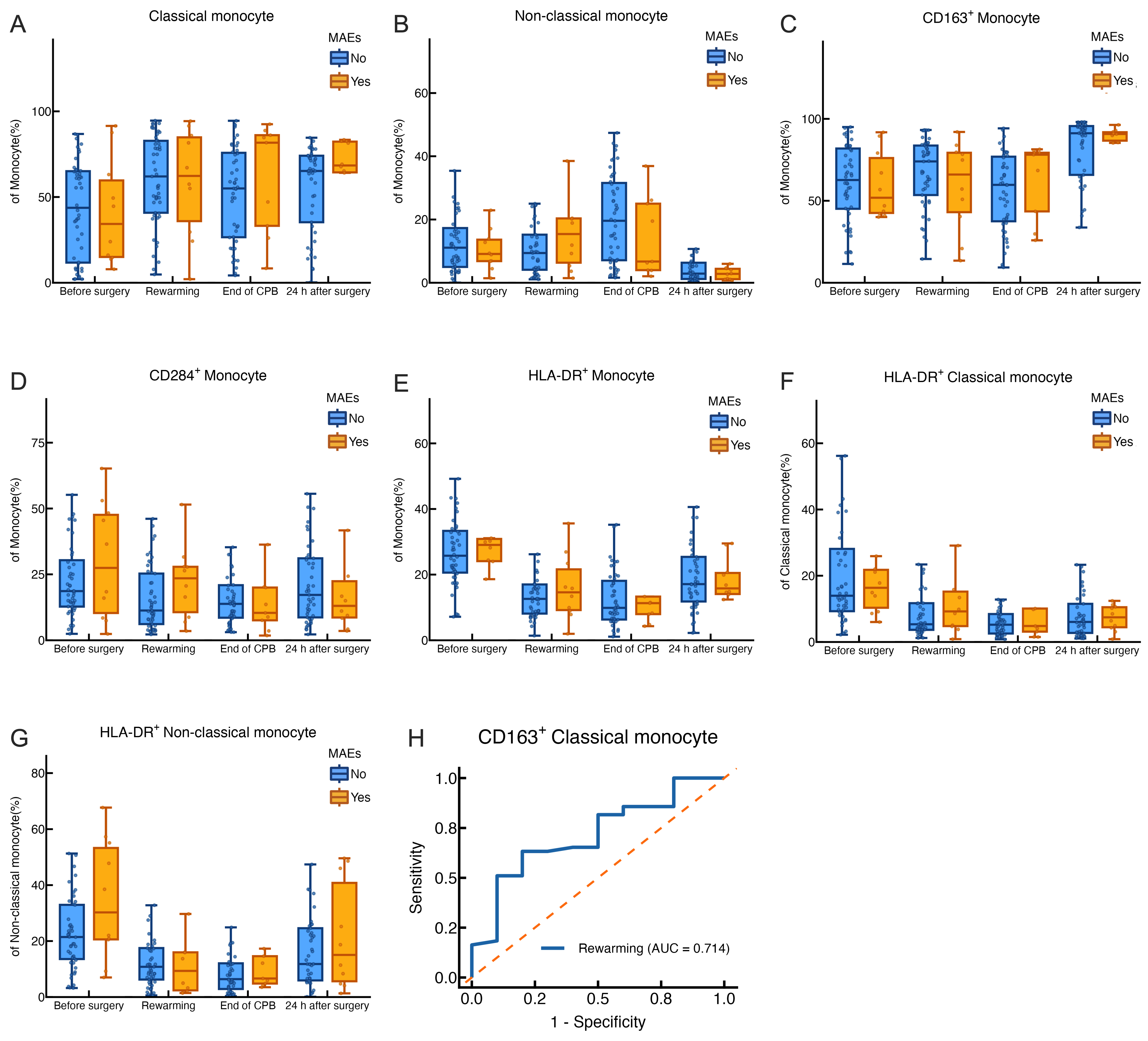


Supplementary Figure S4 (A-G) Comparison of the proportions of different monocyte subpopulations at different times before, during, and after cardiopulmonary bypass (CPB) between the 10 patients who experienced major adverse postoperative events (MAEs) within 30 days after surgery and the 50 patients who did not. (H) Exploratory receiver operating characteristic curve (ROC) describing within-cohort discrimination between patients with and without MAEs based on the indicated monocyte subpopulation at rewarming. The area under the curve (AUC) is shown at the lower right.


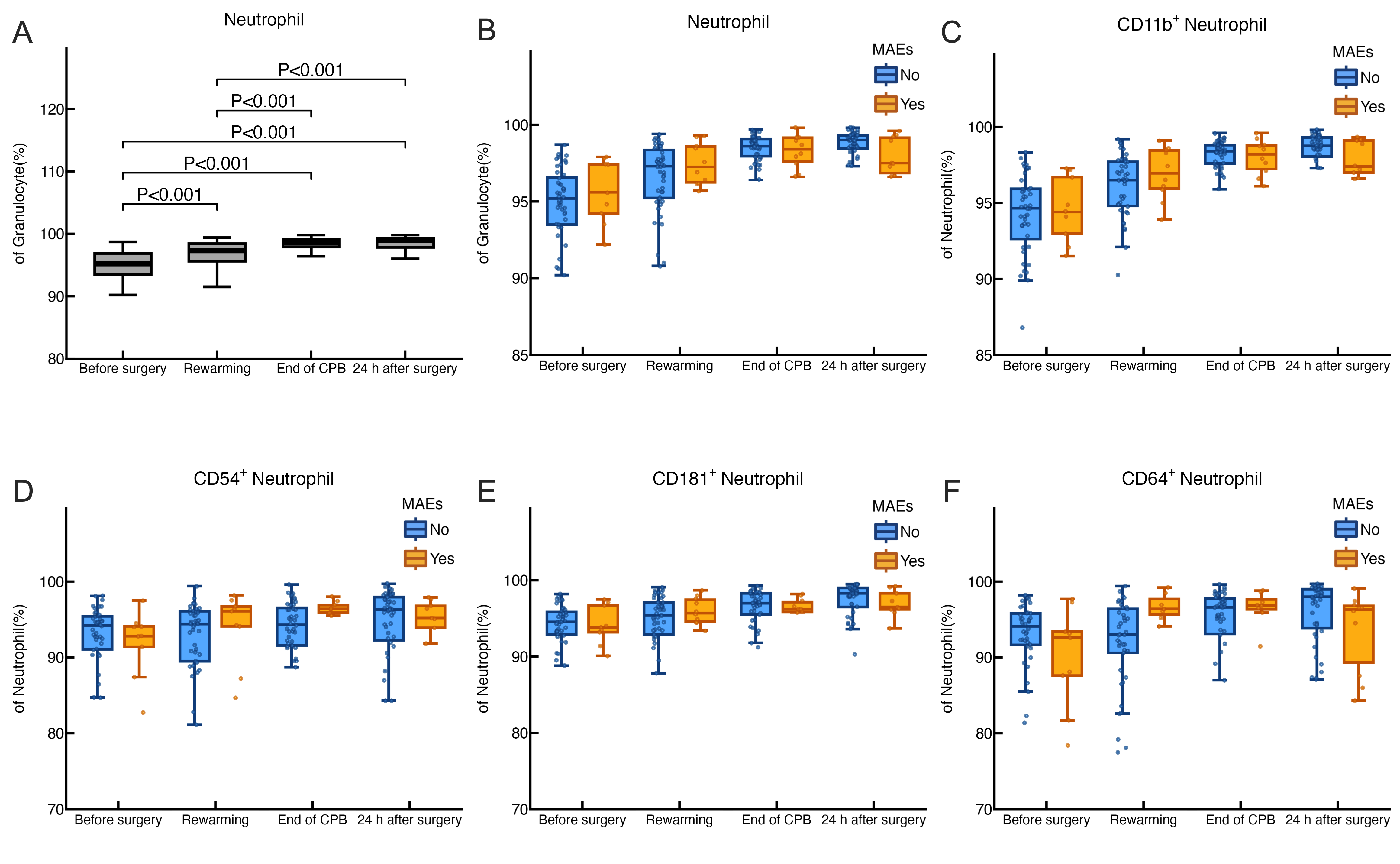


Supplementary Figure S5 (A) Trajectory of the relative abundance of neutrophils at different times before, during, and after cardiopulmonary bypass (CPB). (B-F) Comparison of the proportions of total neutrophils or different neutrophil subpopulations at different times between the 10 patients who experienced major adverse postoperative events (MAEs) within 30 days after surgery and the 50 patients who did not. The y-axes begin at 70–90% to make differences more visible.

Supplementary Table S2 Sensitivity analysis of the associations between immune markers and major adverse postoperative events (MAEs) after additional adjustment for baseline aspartate aminotransferase (AST).

| Immune marker (per 1-SD increase) | Adjusted OR (95% CI) | P value | 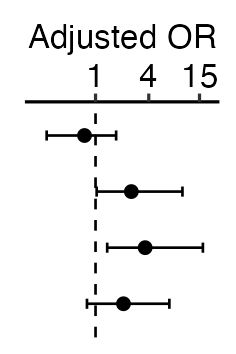 |
| --- | --- | --- | --- |
| CD163⁺ classical monocytes (Rewarming) | 0.75 (0.28–1.71) | 0.500 |  |
| CD284⁺ non-classical monocytes (Rewarming) | 2.54 (1.03–9.62) | 0.042 |  |
| CD274⁺ unswitched memory B cells (End of CPB) | 3.64 (1.36–16.38) | 0.008 |  |
| CD4⁺CD38⁺ T cells (End of CPB) | 2.07 (0.80–6.84) | 0.138 |  |

Models were adjusted for age, baseline estimated glomerular filtration rate (eGFR), preoperative C-reactive protein (CRP), and AST. Immune markers were z-score standardized (per 1-SD increase); ORs therefore represent the change in odds of MAEs per 1-SD increase in marker proportion. Firth penalized logistic regression was applied given the limited number of MAEs.
